# Acute kidney injury caused by traditional Chinese medicinal scorpion: Case report

**DOI:** 10.1097/MD.0000000000048157

**Published:** 2026-03-20

**Authors:** Rui Ma, Beilei Zheng, Jinlin Han, Jingwen Wang, Minghui Cai

**Affiliations:** a First Teaching Hospital of Tianjin University of Traditional Chinese Medicine, Tianjin, China; b National Clinical Research Center for Chinese Medicine, Tianjin, China; c School of Traditional Chinese Medicine, Tianjin University of Traditional Chinese Medicine, Tianjin, China; d School of Pharmacy, University of Nottingham, Nottingham, UK.

**Keywords:** acute kidney injury, medicinal scorpion

## Abstract

**Rationale:**

Taking scorpions (whether through traditional Chinese medicine decoctions containing scorpion or through cooking) carries a potential risk of nephrotoxicity, which deserves people’s attention.

**Patient concerns:**

This article reports a case of a 48-year-old patient who presented with diarrhea and abdominal pain for 3 days and biochemical tests revealed a significant increase in serum creatinine. Based on the clinical inquiry, biochemical examination and imaging report, it is highly suspected that AKI was caused by the medicinal scorpion.

**Diagnoses:**

Acute kidney injury.

**Interventions:**

The treatment involved hydration therapy, along with metabolic promotion and gastric mucosa protection.

**Outcomes:**

Following the treatment, there was a notable reduction in periumbilical distention and pain, with laboratory indicators such as creatinine, urea and uric acid levels returning to normal within 12 days. Follow-ups at 1 week, 1 month, and 3 months after the patient’s discharge revealed no signs of recurrence of abdominal pain or diarrhea.

**Lessons:**

This provided a detailed basis and reference examples for the diagnosis of drug-induced AKI, assisting clinicians in making more accurate and efficient judgments regarding the causes when confronted with similar cases.

## 1. Introduction

Acute kidney injury (AKI) is a clinical syndrome characterized by the rapid loss of renal function, which carries a significant risk of progressing chronic kidney disease and end-stage renal disease.^[1]^ Due to the absence of a specific or effective strategy for treating AKI, it has become a significant global health burden characterized by high morbidity and mortality rates.

According to the latest survey results in 2024, drug-induced AKI is responsible for approximately 14% to 37% of all AKI cases.^[2]^ Certain animals, such as cantharides, centipedes and scorpions, exhibit renal toxicity when used in traditional Chinese medicine, potentially leading to AKI. This article explores the nephrotoxic risks and essential clinical monitoring points associated with the use of animal-based traditional Chinese medicines, illustrated through a case of AKI in a middle-aged male resulting from the consumption of a decoction containing medicinal scorpion. It aims to provide a reference basis for the appropriate clinical use of medicinal scorpion.

## 2. Case report

The patient is a 48-year-old Asian male office worker, measuring 175 cm in height and weighing 70 kg, resulting in a BMI of 22.9. He has no history of smoking, alcohol consumption, or familial diseases. On December 8th, 2024, the patient sought medical attention at the outpatient department of the First Teaching Hospital of Tianjin University of Traditional Chinese Medicine due to hypertension and tinnitus. The doctor prescribed traditional Chinese medicine for treatment, which included Gastrodia, Poria, Salvia Miltiorrhiza, Tangerine Peel, medicinal Pinellia Tuber, Cortex Albiziae, Phellodendri, Cloves stir-fried Six Shenqu and Medicinal Scorpion. On December 9th, the patient exhibited symptoms of diarrhea, characterized by watery stools, periumbilical distending pain, and oliguria, with no apparent underlying cause. On December 13th, the biochemical test upon admission showed that the serum creatinine level was 514.23 μmol/L, which was at least 1.5 times higher than the baseline value, meeting the diagnostic criteria for AKI.

**Figure 1.**
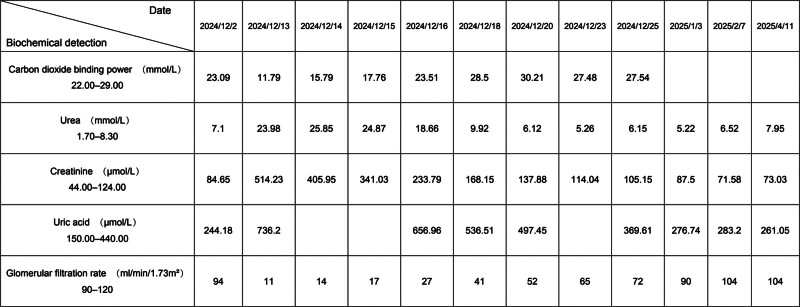
Biochemical detection.

**Figure 2.**
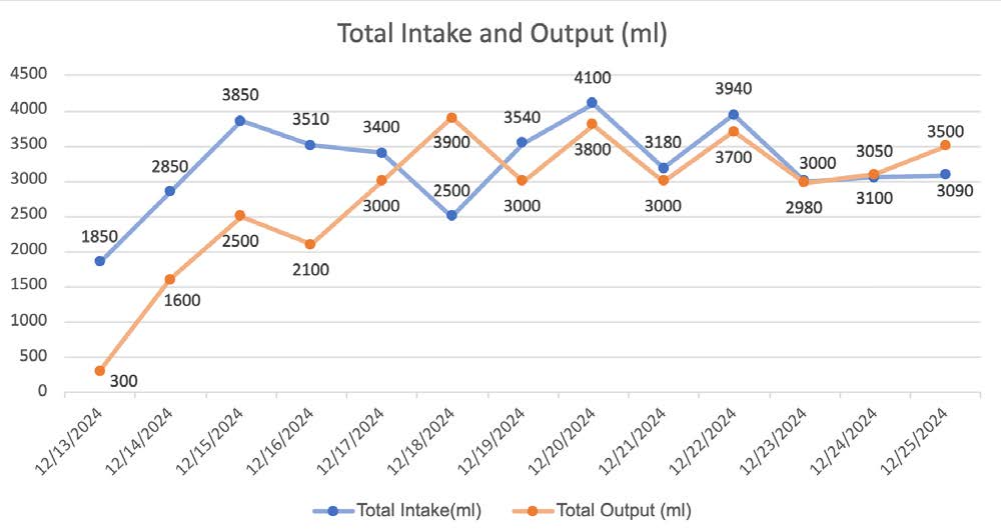
Total intake and output.

Preliminary laboratory results indicated an increase in urea, creatinine, and uric acid levels, alongside a decrease in carbon dioxide binding power, with detailed data presented in Figure 1 and Supplemental Digital Content 1, Supplemental Digital Content, https://links.lww.com/MD/R571. The detailed data of the patient’s total intake and output are shown in Figure 2 and Supplemental Digital Content 2, Supplemental Digital Content, https://links.lww.com/MD/R572.

**Figure 3.**
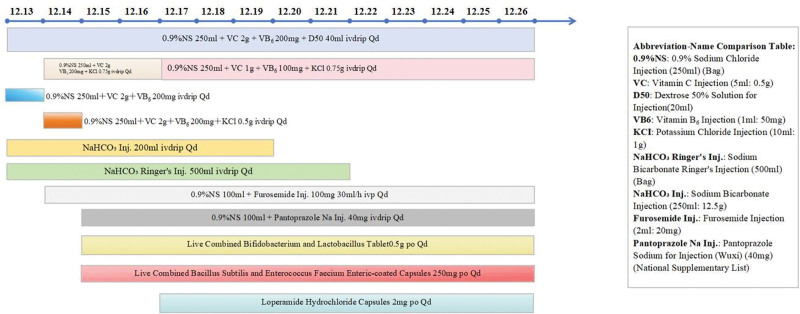
Medication data.

The patient discontinued the traditional Chinese medicine prescription and underwent hydration therapy, which included sodium bicarbonate injection, 0.9% sodium chloride injection, potassium chloride injection, and sodium bicarbonate Ringer injection. Furthermore, metabolic promotion is facilitated through the administration of vitamin C injections, vitamin B_6_ injections, 0.9% sodium chloride injections, and furosemide injections. The gastric mucosa is protected through the injection of pantoprazoles. Details regarding specific usage and dosages are shown in Figure 3 and Supplemental Digital Content 3, Supplemental Digital Content, https://links.lww.com/MD/R572. Following the treatment, there was a notable reduction in periumbilical distention and pain, with creatinine, urea and uric acid levels returning to normal within 12 days.

The patient returned to the hospital for a follow-up visit on January 3rd, 2025. The abdominal pain symptoms had resolved, tinnitus symptoms had not recurred, and both defecation and urination were normal. The biochemical examination revealed that the levels of blood creatinine, urea, uric acid, and other indicators were within the normal range. The patient continued to take the original Chinese medicine prescription, which included Gastrodia, Poria, Salvia Miltiorrhiza, Tangerine Peel, medicinal Pinellia Tuber, Cortex Albiziae, Phellodendri, Cloves stir-fried Six Shenqu, for the purpose of blood pressure control. The patient attended follow-up visits on February 7th and April 11th. On both occasions, urinalysis and biochemical examinations indicated that serum creatinine, urea, uric acid, and other relevant parameters remained within normal limits. The patient reported no discomfort or adverse symptoms, continued with the original Chinese herbal prescription, and maintained satisfactory blood pressure control.

## 3. Discussion

**Figure 4.**
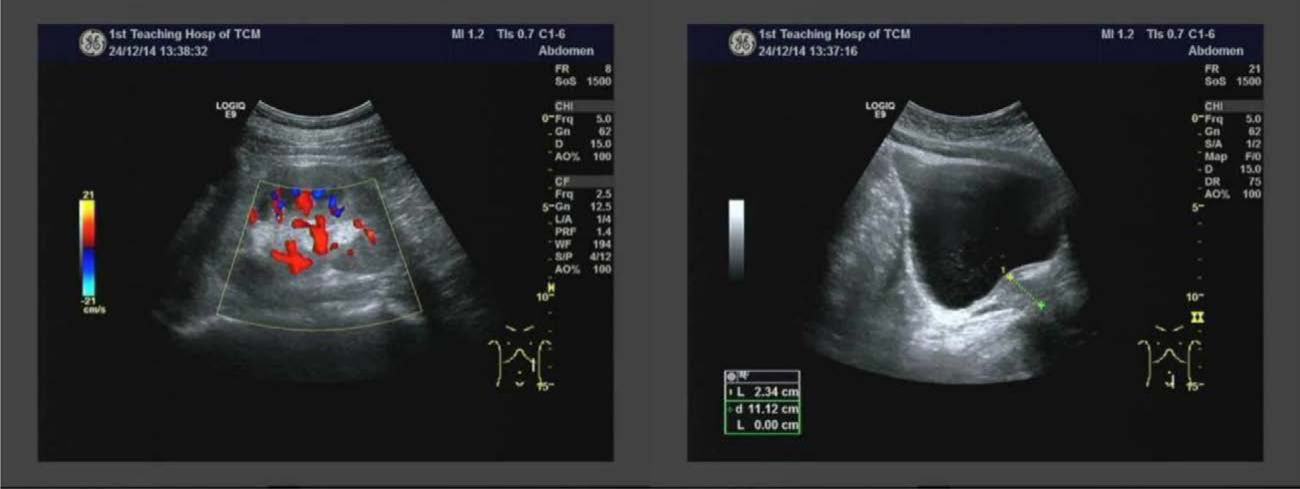
Urinary color doppler image.

AKI is one of the most prevalent critical clinical conditions. The diagnostic criteria for AKI must satisfy at least one of the following conditions^[1]^: (1) an increase in serum creatinine of ≥26.5 μmol·L^−1^ (0.3 mg·dL^−1^) within 48 hours; (2) an increase in serum creatinine of more than 1.5 times the baseline value within the past 7 days; (3) a urine output of 10.5 mL·kg^−1^·h^−1^ for 6 hours. The patient sought medical consultation at the hospital due to experiencing abdominal pain. Biochemical examination revealed significant abnormalities in creatinine, urea, uric acid, and other indicators (Fig. 1). Abdominal and gastrointestinal color Doppler ultrasonography, color echocardiography, and high-resolution spiral CT plain scans of the chest revealed was normal. However, the urinary color Doppler examination (shown in Fig. 4) indicated thickening of the parenchyma in both kidneys, accompanied by enhanced echogenicity. Based on the patient’s urinary color Doppler results, serum creatinine levels (Fig. 1), and total fluid intake and output (Fig. 2), the patient fulfills the diagnostic criteria outlined in (2) and (3) and can thus be diagnosed with AKI.

The results of biochemical examinations, in conjunction with urinary color Doppler imaging, confirmed the presence of AKI. AKI can be classified into 3 primary categories-prerenal, nephrogenic, and postrenal-based on the location of the lesion and its underlying cause.^[3]^ Analysis of the patient’s detailed total fluid intake and output revealed no evidence of prerenal causes associated with hypovolemia. Furthermore, renal B-mode ultrasonography clearly indicated the absence of postrenal diseases, such as obstruction from urinary stones and tumors. Findings from routine blood tests largely excluded the possibility of infection; however, the elevated eosinophil and basophil counts indicated a potential allergic reaction. Results from comprehensive immunological testing, rheumatologic panels, and autoantibody screening ruled out immune-related disorders. When considered alongside tubular urine biochemical indices, these findings collectively supported the diagnosis of nephrogenic tubular injury, primarily attributed to drug exposure.

A comprehensive review of the patient’s treatment history revealed that the patient has consistently taken Chinese herbs, including Gastrodia, Poria, Salvia Miltiorrhiza, Tangerine Peel, medicinal Pinellia Tuber, Cortex Albiziae, Phellodendri, Cloves stir-fried Six Shenqu, to manage hypertension since May 11th, 2024. Blood pressure has been well controlled under this herbal treatment, maintained at approximately 130/80 mm Hg, with no adverse reactions reported. The patient sought medical attention at the outpatient department of the hospital on December 8th, 2024 due to hypertension and tinnitus. While maintaining the original traditional Chinese medicine prescription for hypertension management, the doctor added an animal medicine, medicinal scorpion, to address the tinnitus. Given that the patient’s hypertension was well controlled and the tinnitus symptoms were mild, AKI from these causes was excluded.

After the patient was admitted to the hospital, he reported that he had maintained a normal diet recently, had not consumed any new foods or health care products, and followed a regular work and rest schedule. The patient had no prior history of kidney disease, and after excluding other potential causes of AKI, it was highly suspected that the AKI was induced by medicinal scorpion based on the temporal correlation. This batch of medicinal scorpions has passed the quality inspection report and is considered a qualified product. And after further inquiry, it was ascertained that the patient’s method of decocting the traditional Chinese medicine was correct. Therefore, it can be ruled out that this adverse reaction was caused by improper processing of the medicinal centipede.

**Figure 5.**
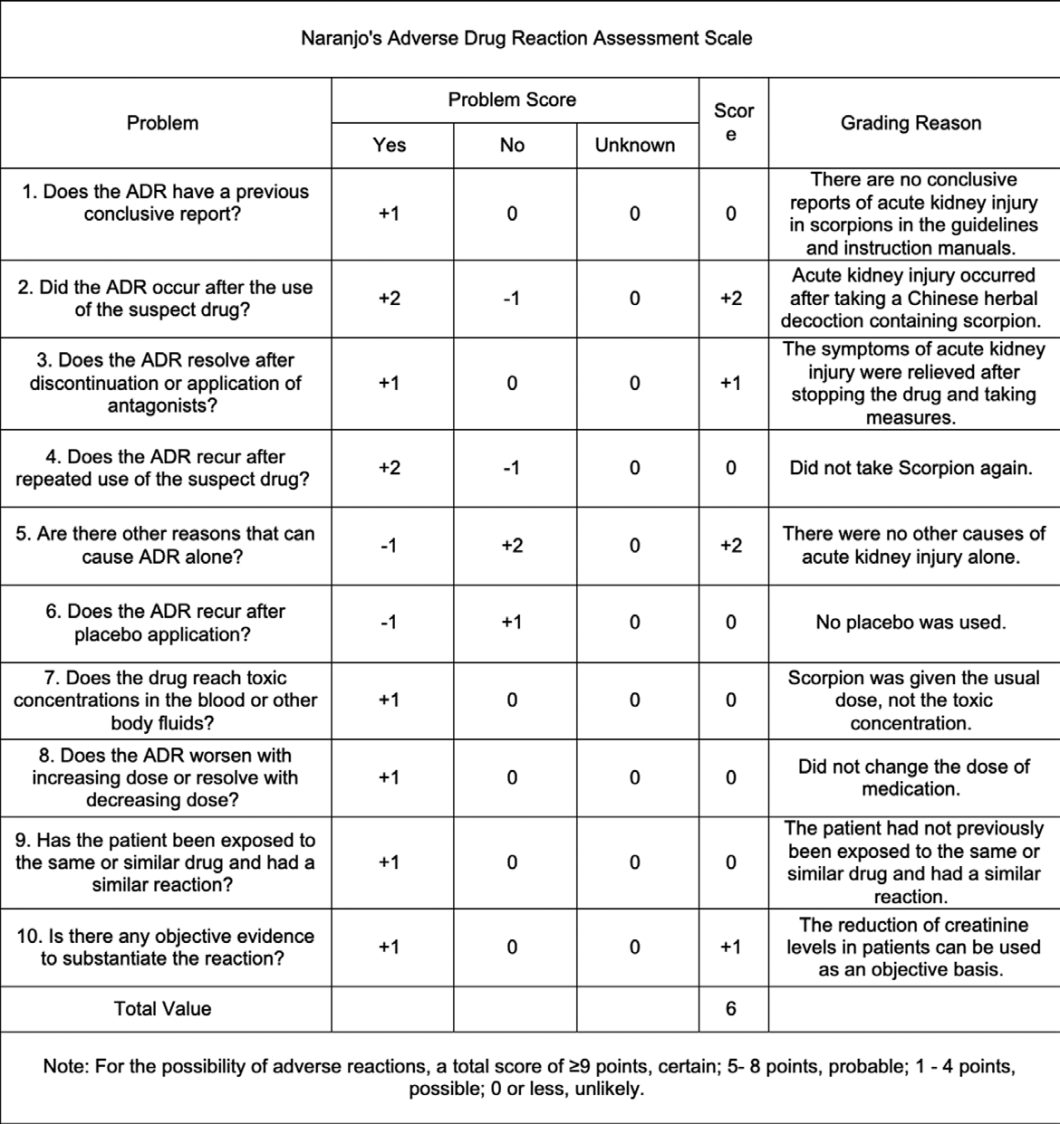
Naranjo’s adverse drug reaction assessment scale. ADR = adverse drug reaction.

According to Naranjo’s Adverse Drug Reaction Assessment Scale (Fig. 5), the score was 6, indicating a probable relationship. Consequently, the patient discontinued the traditional Chinese medicine prescription during hospitalization. After discharge, the patient continued to adhere to the original traditional Chinese medicine prescription for blood pressure management and consistently attended follow-up visits, with no further adverse reactions reported.

The Chinese scorpion, also known as Quanxie, is a valuable herbal remedy in traditional Chinese medicine, boasting a medicinal history that spans over 1000 years. The 2020 edition of the Chinese Pharmacopoeia defines the whole scorpion as *Buthus martensii* Karsch, which is known to be poisonous.^[4]^ Recent pharmacological studies have demonstrated that scorpion possesses significant antitumor, antiepileptic, anticonvulsive, anticoagulation, and antibacterial effects, in addition to its analgesic, anti-inflammatory, pro-growth, and immune-enhancing activities.^[5]^ However, there is a paucity of research regarding the adverse reactions associated with its use.

Reports on nephrotoxicity associated with the ingestion of scorpions – whether through traditional decoctions containing scorpions or direct culinary consumption – are relatively scarce; however, several case reports provide supporting evidence. For instance, an article published in CHEST described a 64-year-old male who developed acute toxic myocarditis, hepatic injury, and AKI, which ultimately led to multiple organ failure after long-term oral intake of scorpions for the relief of arthritis.^[6]^

Both scorpion and centipede belong to arthropods, and they share high commonalities in medicinal material properties and toxicology: both possess specialized venom glands and secrete active toxins.^[7]^ The main components of their toxins are mostly thermostable neurotoxic peptides, which induce nephrotoxicity by acting on ion channels such as sodium and potassium channels.

Previous reports have documented cases of rhabdomyolysis, myoglobinuria, and AKI following the oral ingestion of medicinal liquor prepared with processed centipedes.^[8]^ Furthermore, more definitive evidence supports the oral nephrotoxicity of cantharides, another arthropod-derived medicinal material. Patients who ingested teas or preparations containing cantharidin subsequently developed AKI.^[9]^ Additionally, cantharidin, the principal bioactive component of cantharides, has been experimentally confirmed to exert direct cytotoxic effects on renal tubular epithelial cells, ultimately resulting in acute tubular necrosis.^[10]^ Collectively, these cases suggest that orally administered toxic traditional medicines derived from arthropods can lead to severe renal injury. Consequently, these findings offer indirect yet compelling evidence supporting the potential nephrotoxicity associated with scorpion ingestion. Given the common mechanisms of toxicity, a cautious approach is warranted, which includes avoiding the combined administration of these arthropod-based materials and tailoring therapeutic regimens to mitigate the risk of cumulative renal injury.

In traditional Chinese medicine, scorpions are often prescribed with other herbal materials and are administered in various dosage forms, including decoctions, pills, and powders. According to the Pharmacopoeia of the People’s Republic of China, the standardized processing method for scorpions includes a “purification” procedure, during which the animals are boiled in water until they become rigid and then dried.^[11]^ This method typically inactivates or diminishes toxic constituents through the application of heat.^[12]^ Nevertheless, proteomic and electrophysiological investigations have demonstrated that even pharmacopeia-processed scorpion materials still contain 22 full-length and 44 truncated thermostable potassium channel-modulating toxins, which at the functional level remain capable of significantly blocking human Kv1 and hERG channels.^[13]^ Importantly, even trace amounts of these residues may be sufficient to provoke allergic reactions or renal injury. Similar integrative approaches that combine omics with functional analyses have been utilized in various studies of scorpion venom. For instance, mechanistic investigations have demonstrated that the scorpion peptide HsTx2 exerts neurological effects through the circRNA/miRNA signaling axis, thereby emphasizing the stability and persistence of venom-derived peptide bioactivity in vivo.^[14]^ Furthermore, additional research has identified degradation peptides that retain activity even in heat-processed scorpions, which may explain their immunomodulatory and potential toxicological effects.^[15]^ Consequently, these findings provide robust scientific evidence at the molecular level supporting the potential nephrotoxicity of orally administered scorpions.

The unique pharmacological components and diverse biological activities of scorpions offer new insights for the treatment of various diseases. However, research on their pharmacokinetics and toxicology is still inadequate, and clear guidelines for dosage control and compatibility contraindications in clinical use are lacking. Therefore, a careful risk assessment is essential when utilizing traditional Chinese medicine formulations that contain animal-derived medicinal materials.

In the future, establishing an Electronic Medication Information Card could assist clinicians during the prescription process by integrating patients’ medication histories, allergy records, and other relevant clinical data. Such a report would generate real-time alerts for potential drug interactions or contraindications, thereby supporting informed decision-making and promoting rational and safe medication practices. Furthermore, it is essential to deepen research on the toxicity mechanisms of medicinal scorpions, systematically analyze the target sites of their active components, establish the dose–toxicity relationship, and elucidate the metabolic pathways. For animal-derived medicinal materials, it is essential to develop rapid detection technologies to monitor the residues of toxic components and to standardize processing techniques to mitigate associated risks.

It is essential to develop a consensus on the use of nephrotoxic animal-derived Chinese medicines, clearly defining contraindicated populations, such as individuals with renal insufficiency, and implementing effective monitoring strategies. Additionally, strengthening patient medication education is crucial to prevent blind usage of these treatments. Through multidisciplinary collaboration and evidence-based medical research, it is possible to maximize the avoidance of nephrotoxicity risks while preserving the efficacy of traditional Chinese medicine. This approach facilitates a scientific application of the principle of “using poison to attack poison.”

## 4. Conclusion

This case report describes a middle-aged man who experienced AKI due to medicinal scorpion in traditional Chinese medicine decoctions. It broadens the etiological spectrum of drug-induced AKI, explicitly highlights the renal toxicity associated with medicinal scorpion, and offers critical insights into the potential risks of animal-based Chinese herbal medicines, such as those containing scorpion. This patient systematically eliminated risk factors through a rigorous examination process and consultations, ultimately clarifying the causal relationship between scorpion exposure and AKI. This provided a detailed basis and reference examples for the diagnosis of drug-induced AKI, assisting clinicians in making more accurate and efficient judgments regarding the causes when confronted with similar cases.

## Author contributions

**Conceptualization:** Minghui Cai, Rui Ma, Beilei Zheng, Jinlin Han.

**Data curation:** Beilei Zheng, Jinlin Han.

**Formal analysis:** Minghui Cai, Rui Ma, Beilei Zheng.

**Methodology:** Minghui Cai, Beilei Zheng, Jinlin Han, Jingwen Wang.

**Project administration:** Minghui Cai, Rui Ma.

**Software:** Beilei Zheng, Jinlin Han, Jingwen Wang.

**Visualization:** Beilei Zheng, Jinlin Han, Jingwen Wang.

**Supervision:** Minghui Cai, Rui Ma, Jinlin Han.

**Validation:** Minghui Cai, Beilei Zheng, Jinlin Han.

**Writing – original draft:** Rui Ma, Beilei Zheng, Jinlin Han, Jingwen Wang.

**Writing – review & editing:** Rui Ma, Beilei Zheng, Jinlin Han.

## Supplementary Material




