# Supplementary figures and images for: Acute kidney injury caused by traditional Chinese medicinal scorpion: Case report
Source: Medicine (Baltimore). 2026 Mar 20;105(12):e48157. doi: 10.1097/MD.0000000000048157 (PMC13008159; doi:10.1097/MD.0000000000048157)

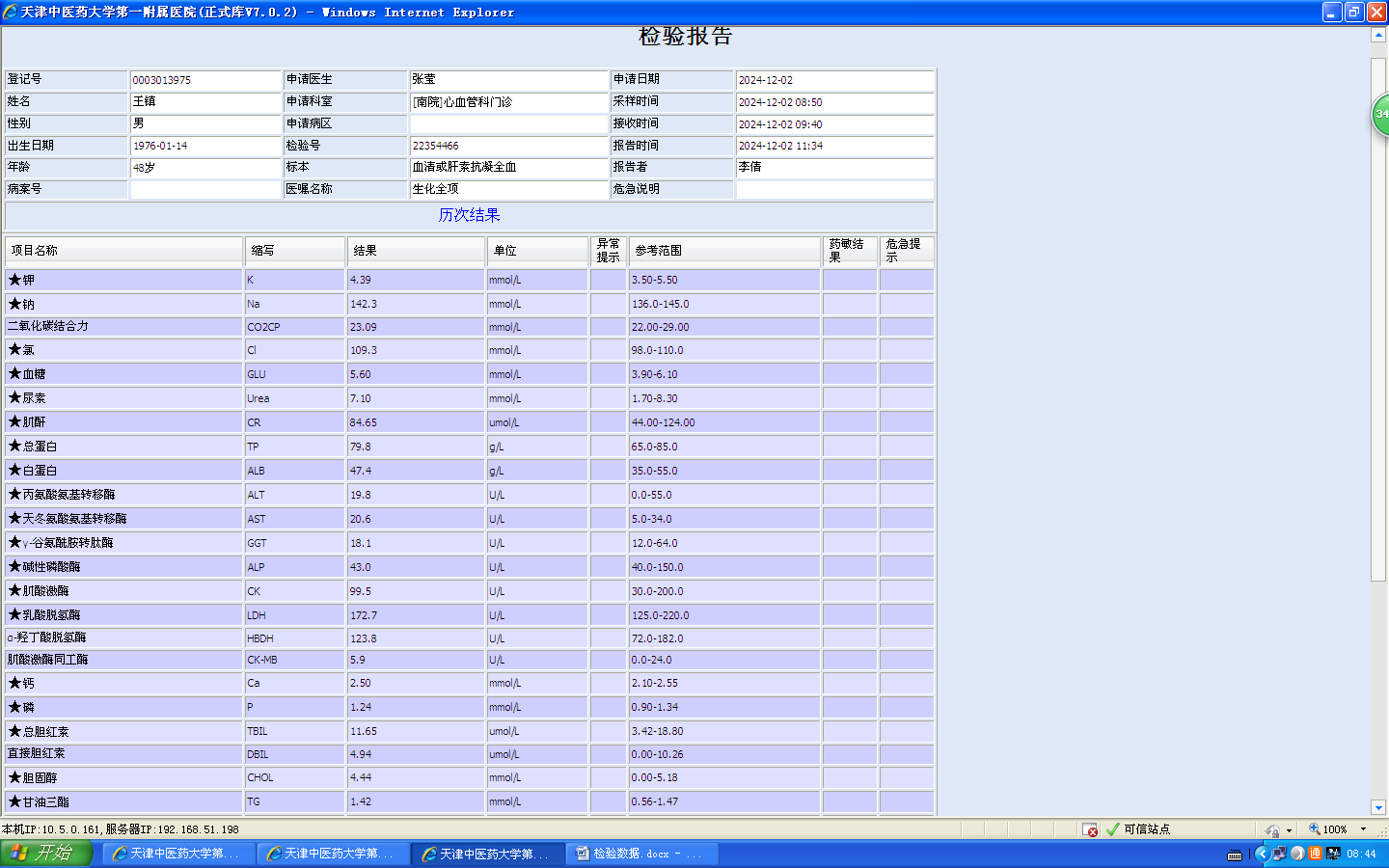


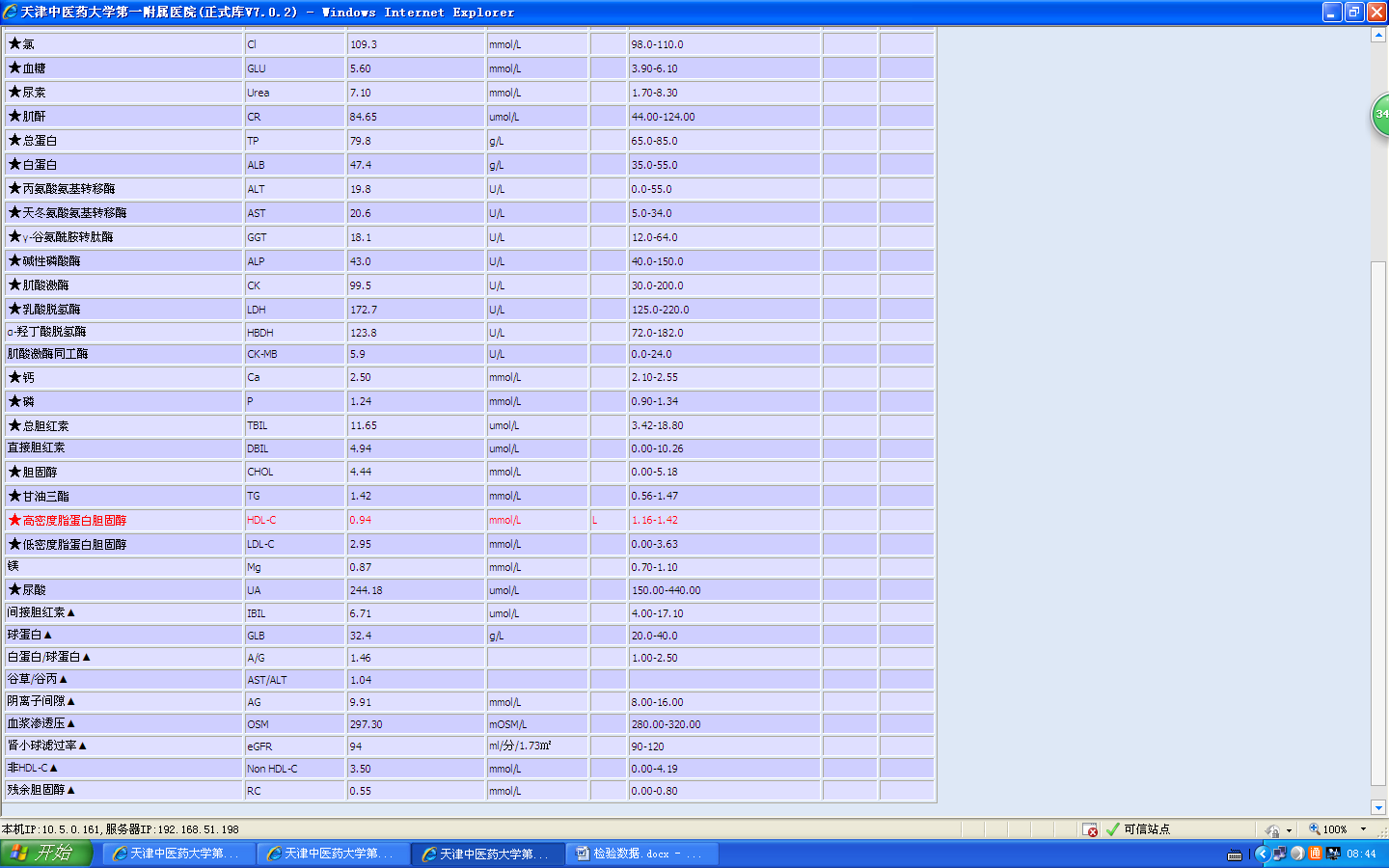


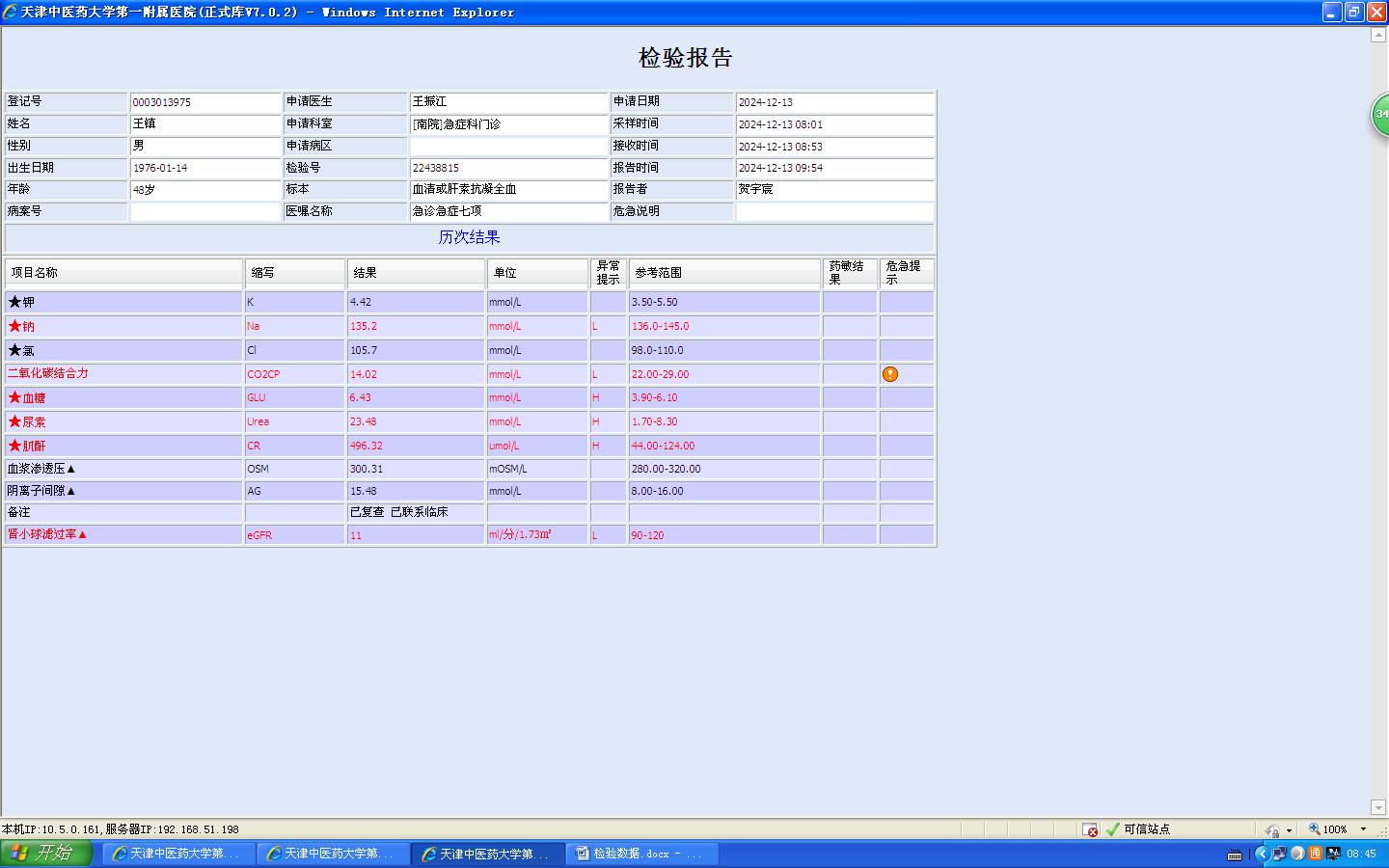


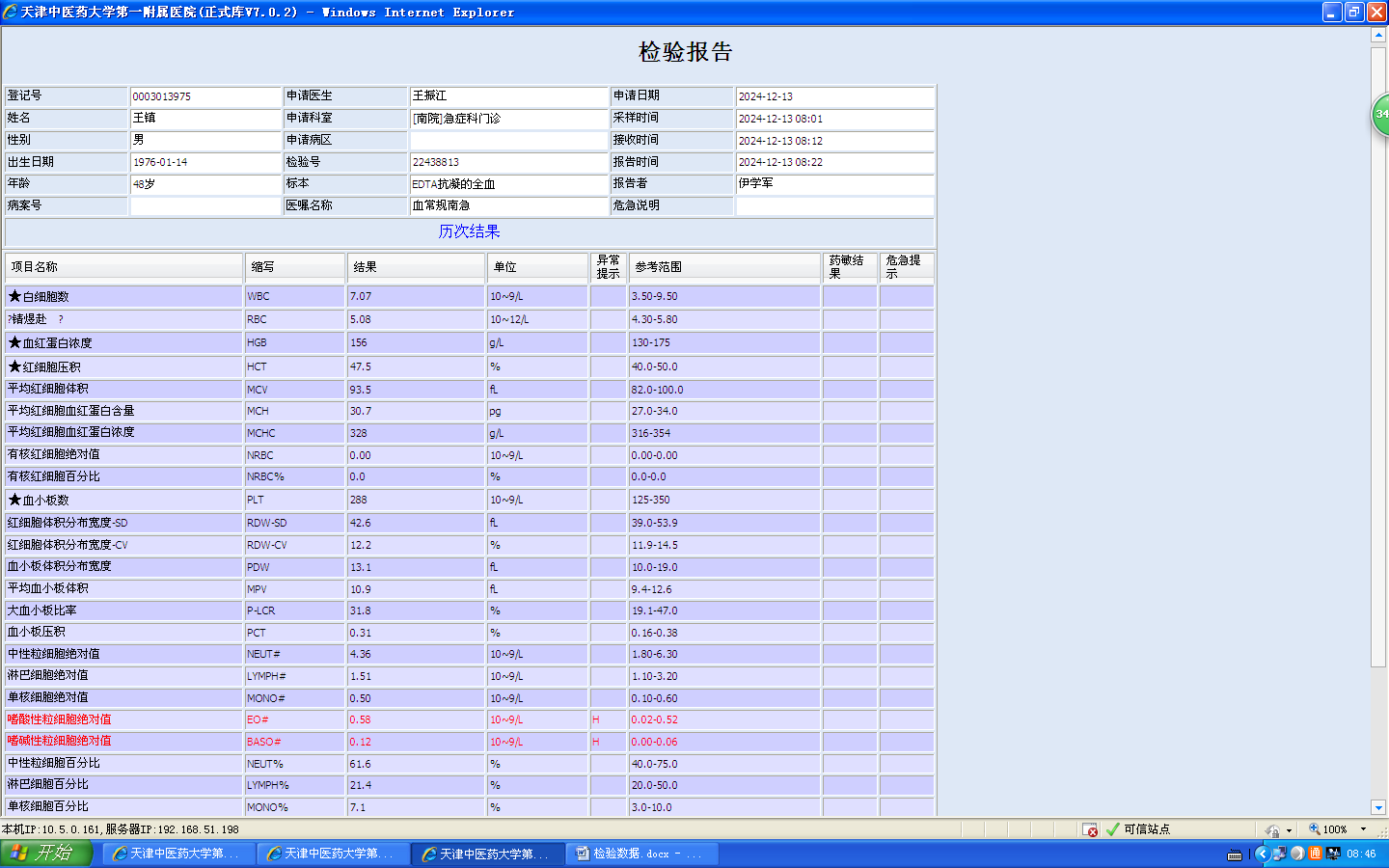


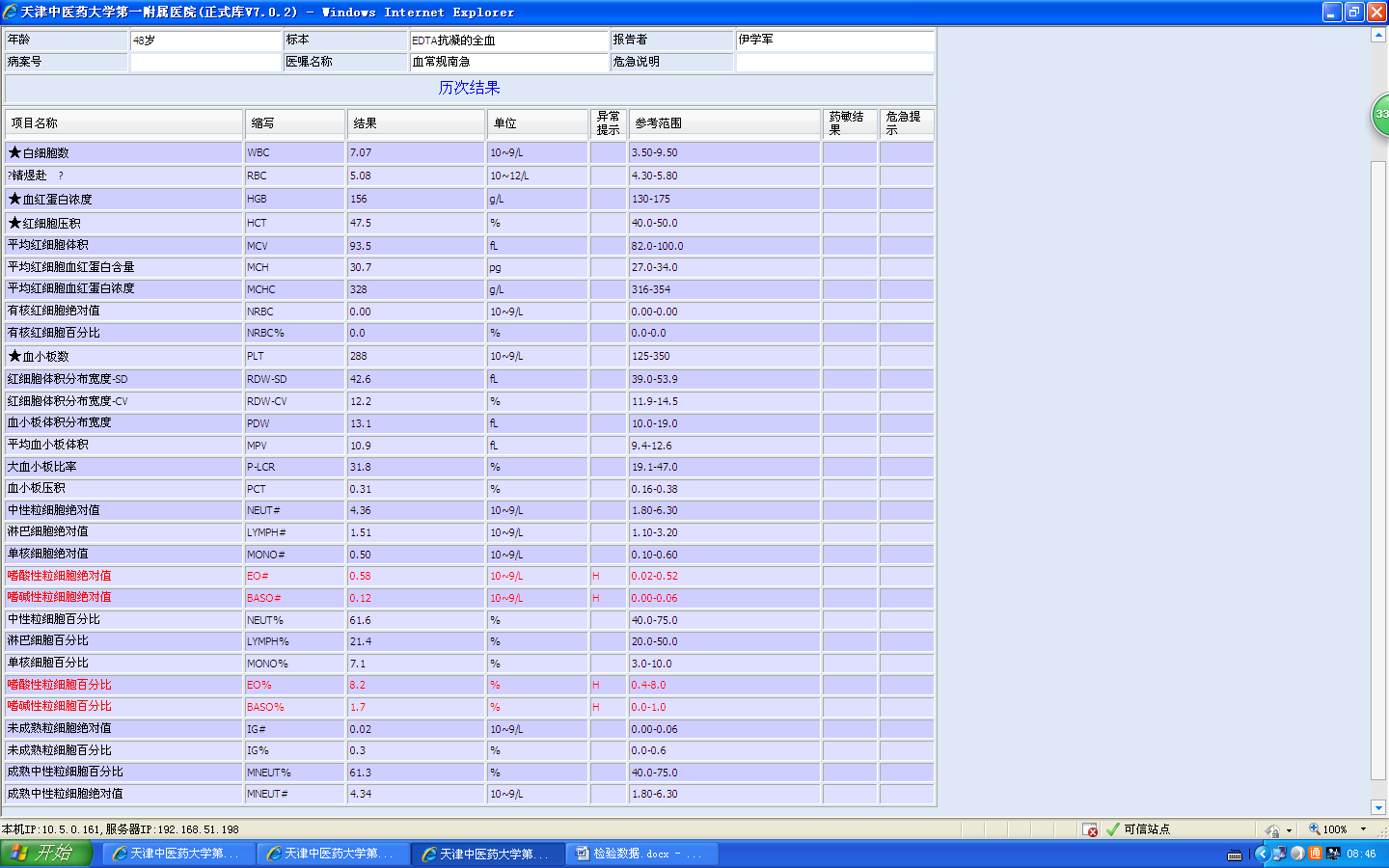


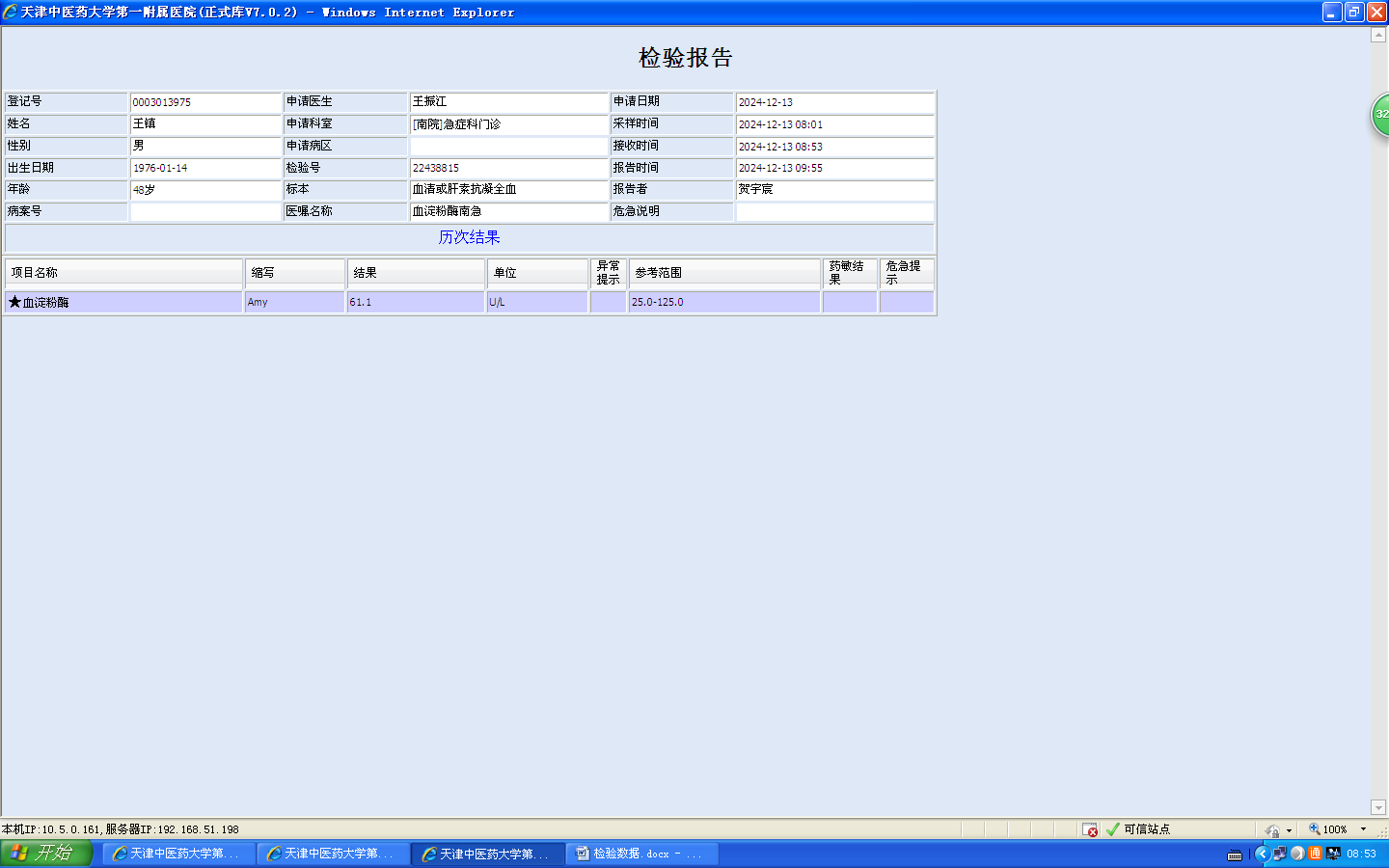


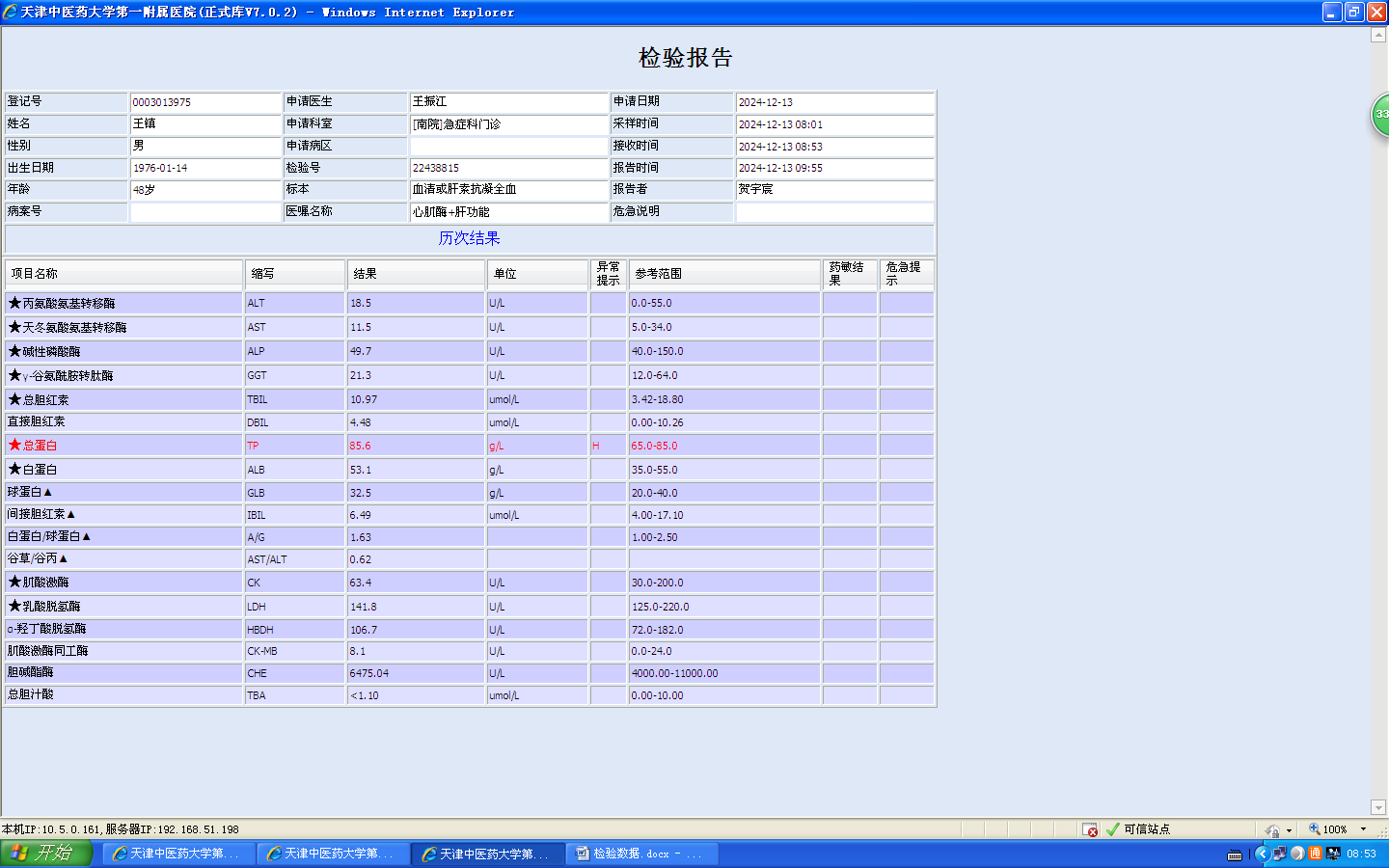


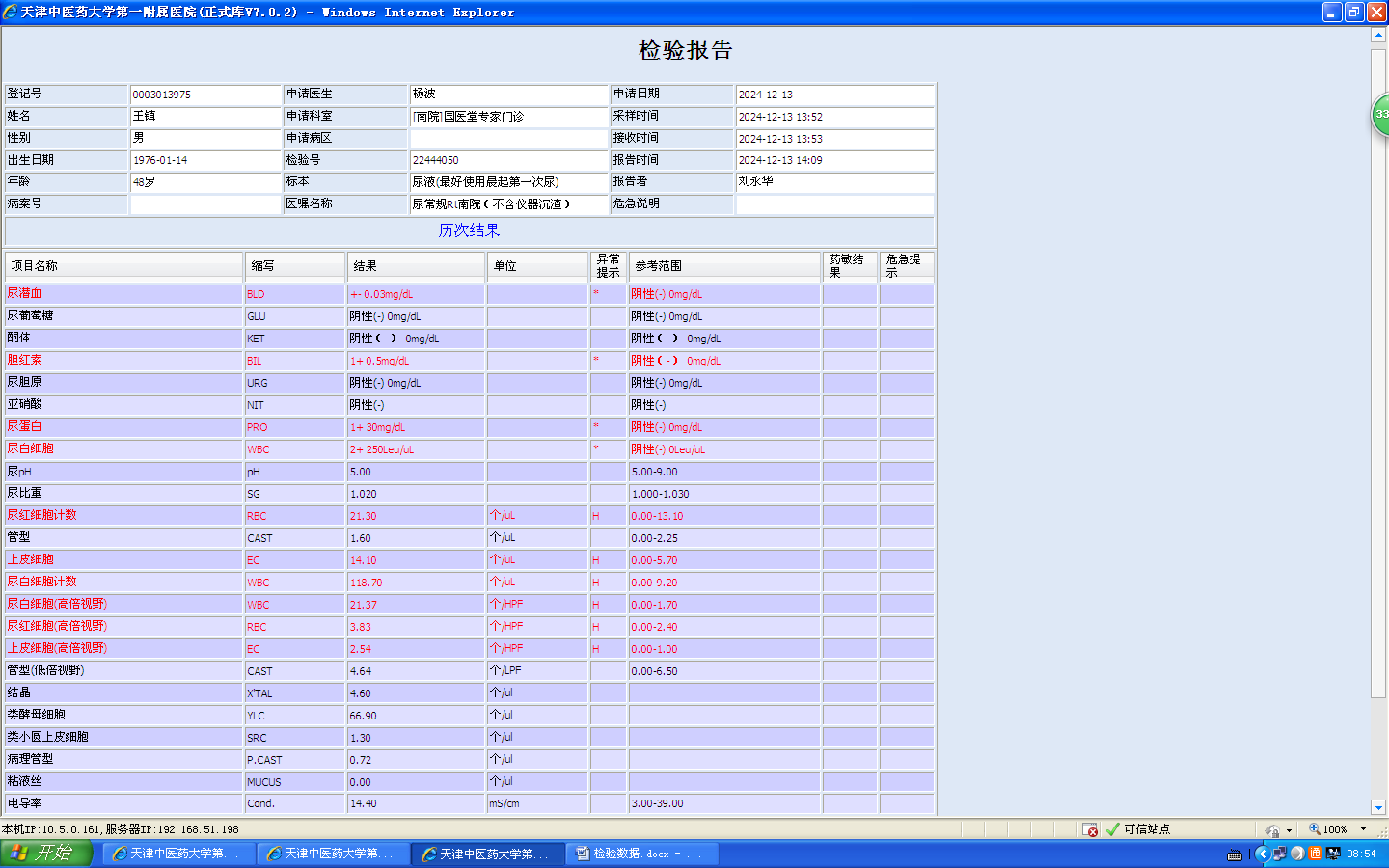


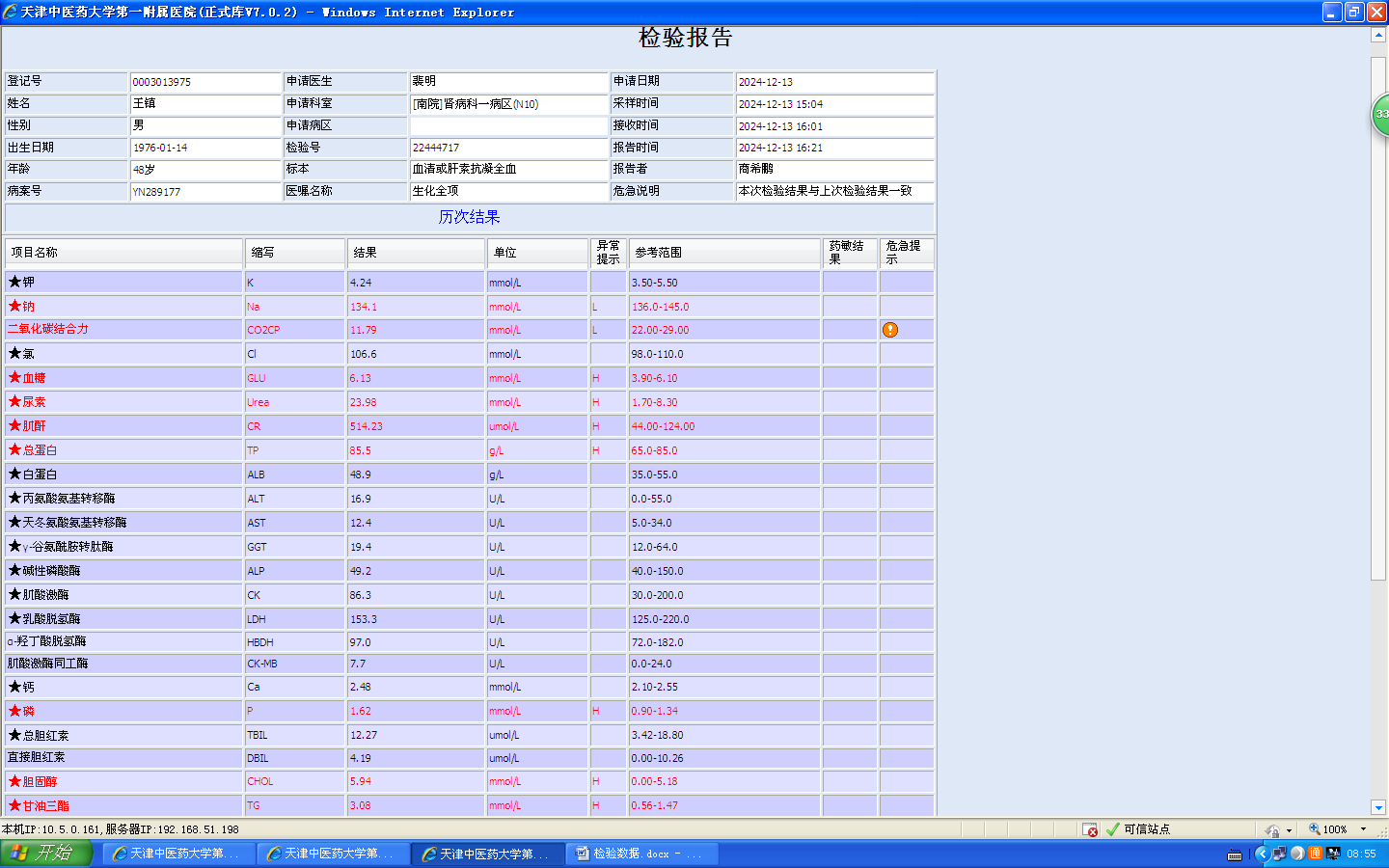


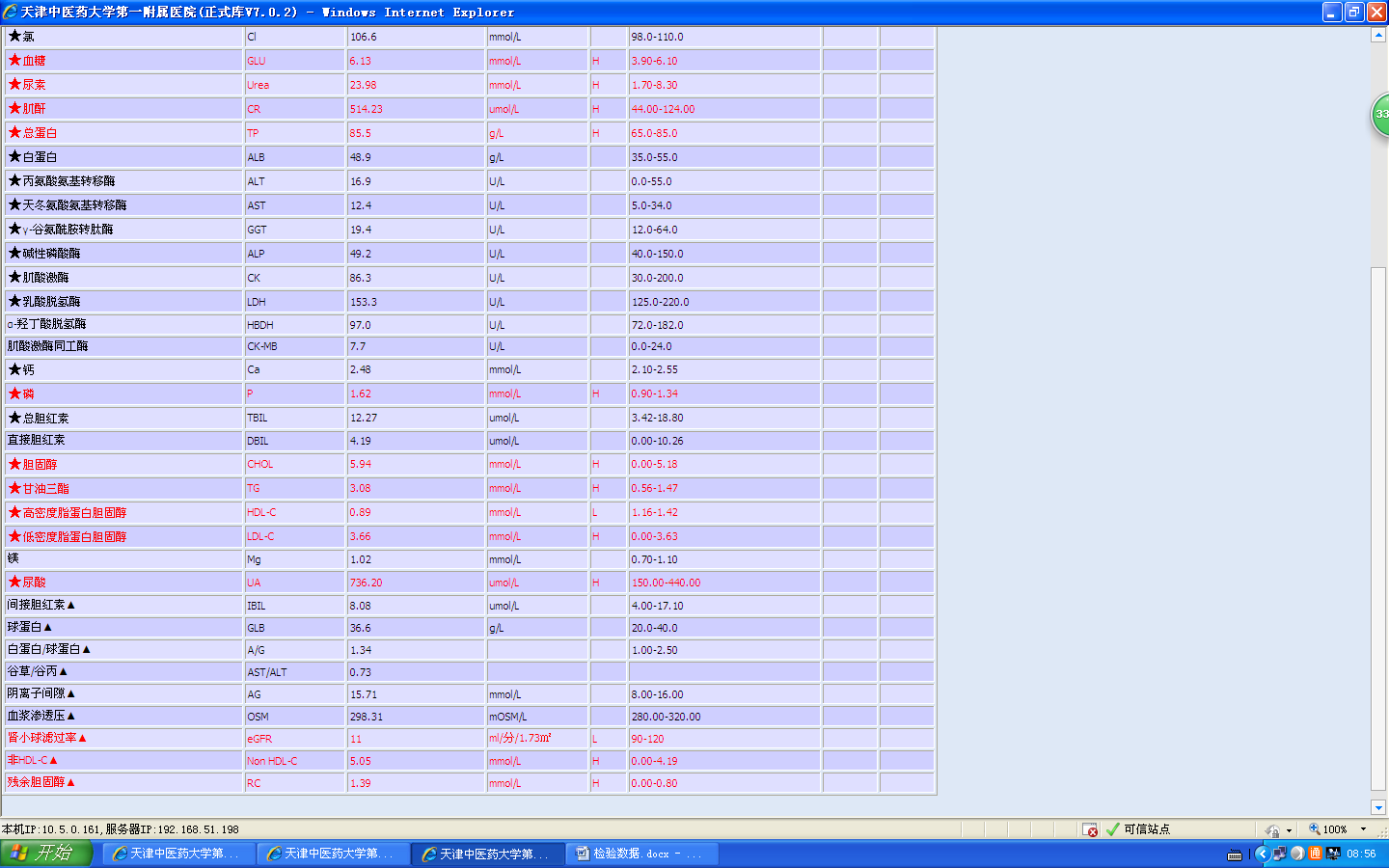


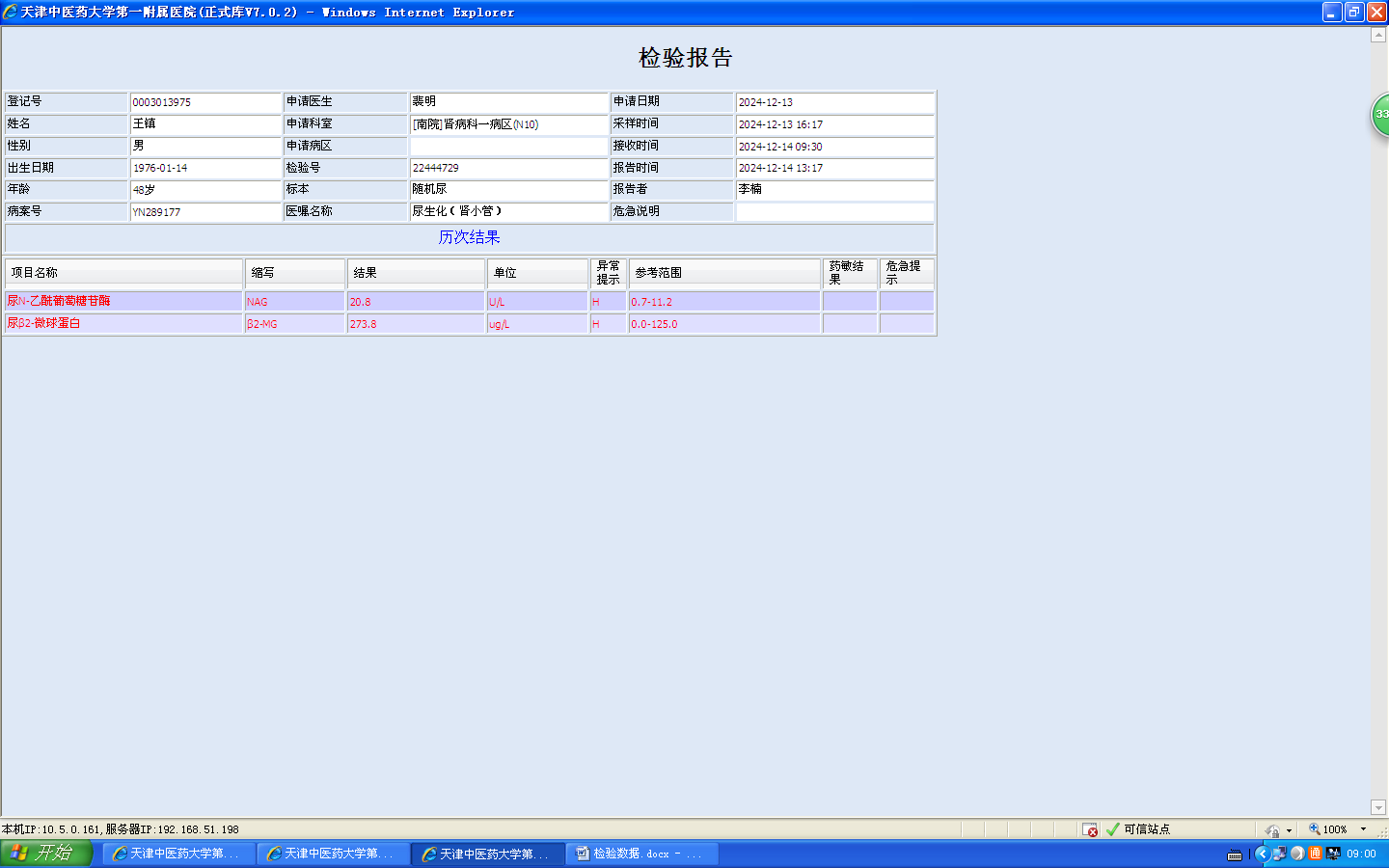


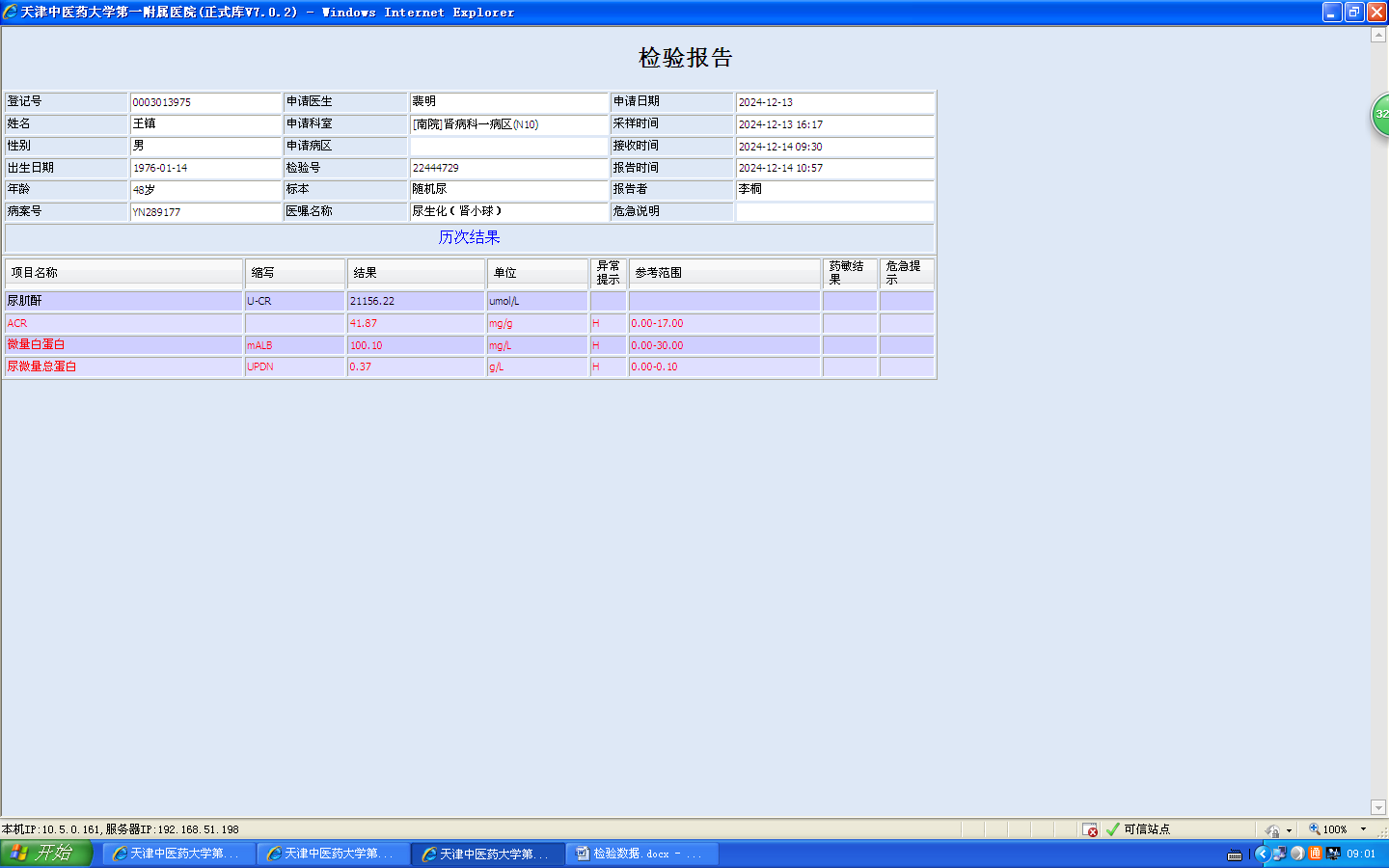


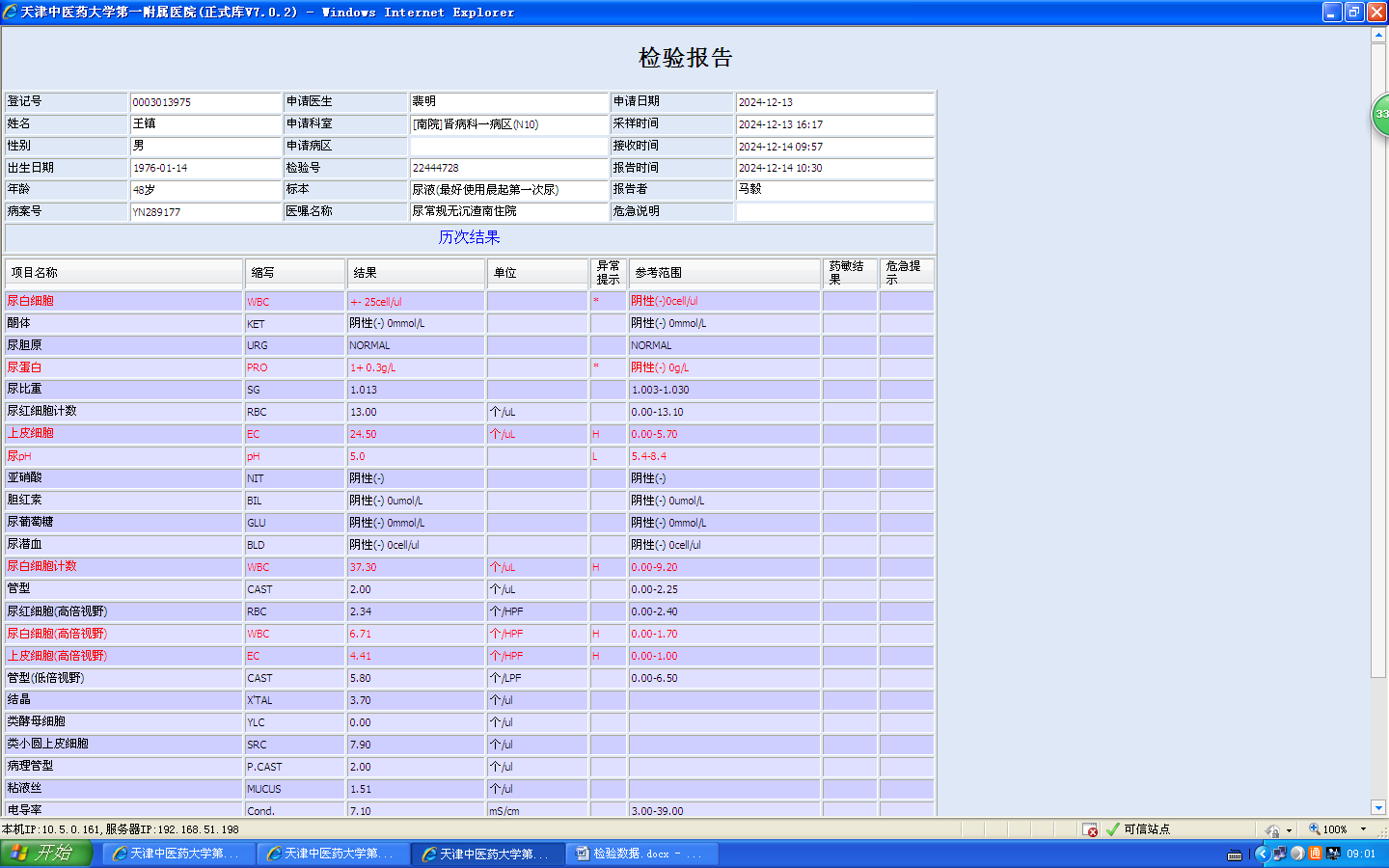


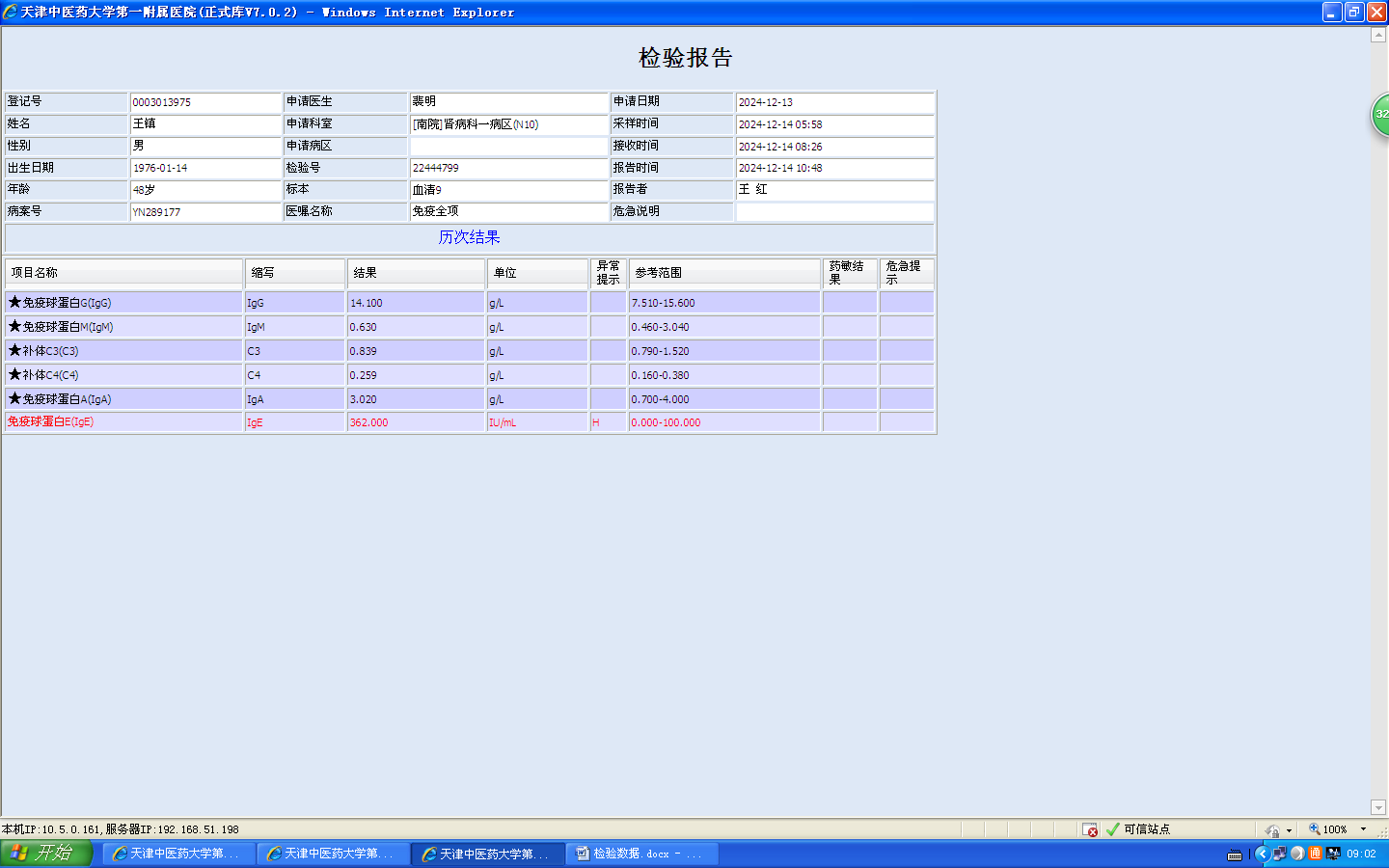


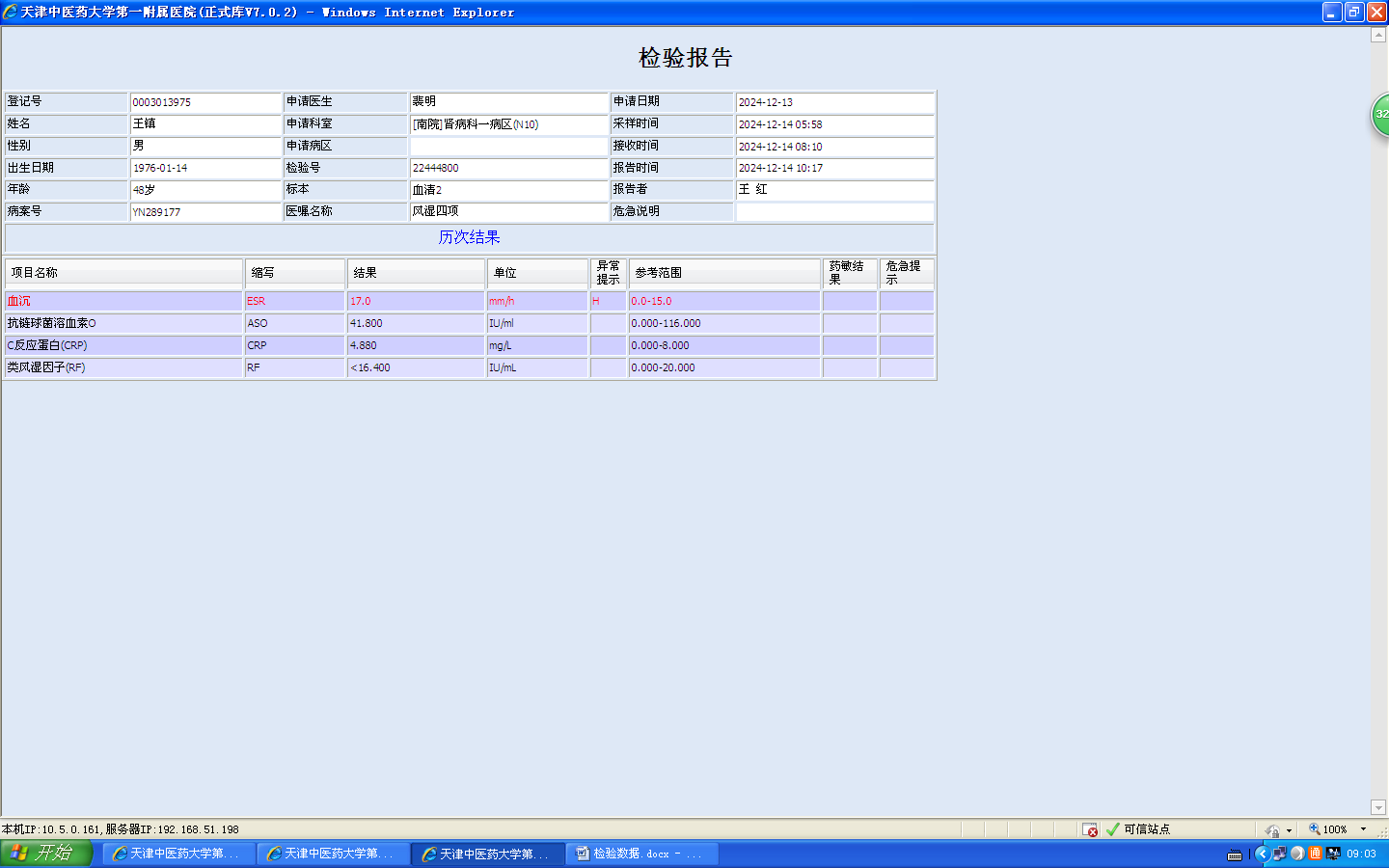


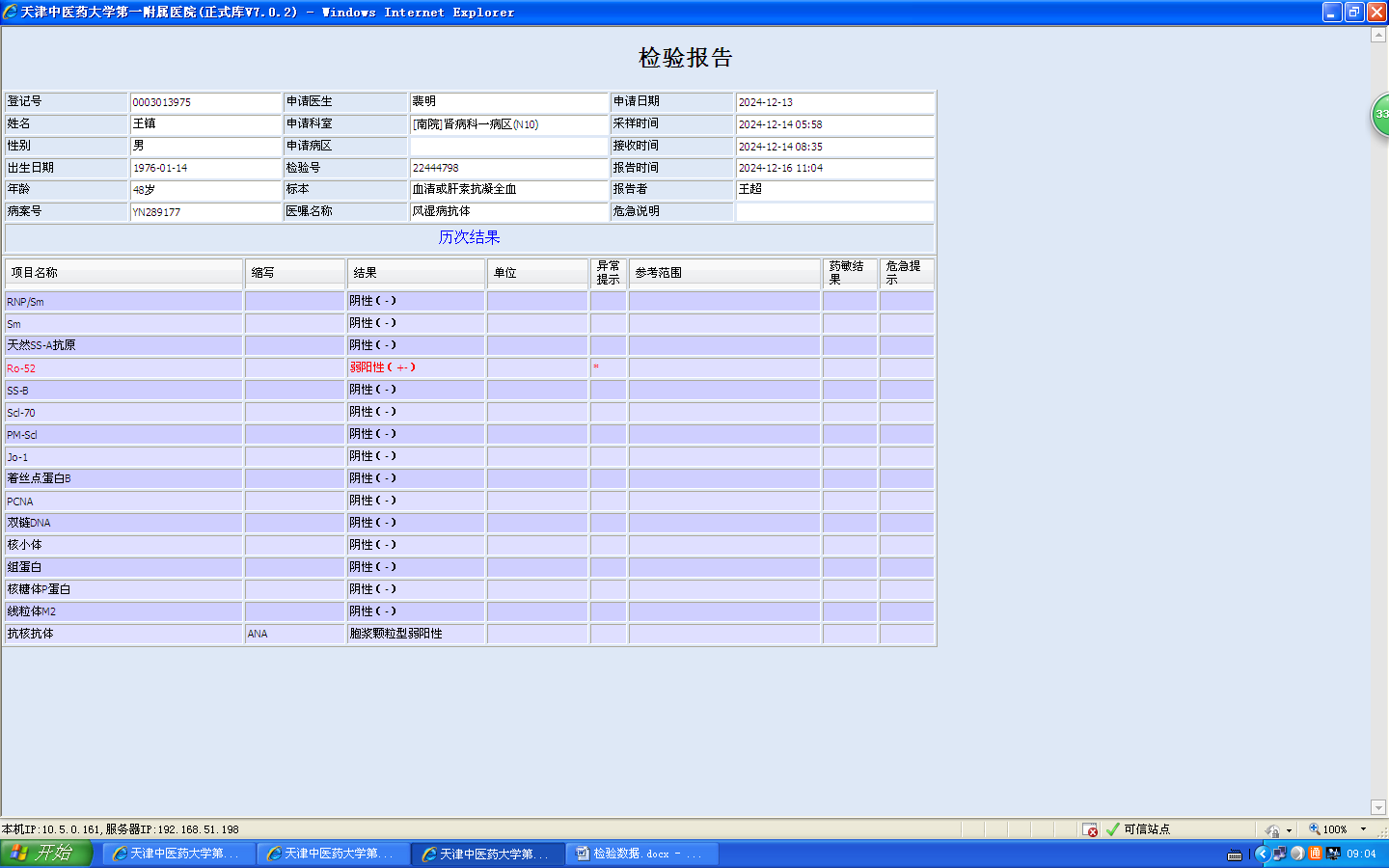


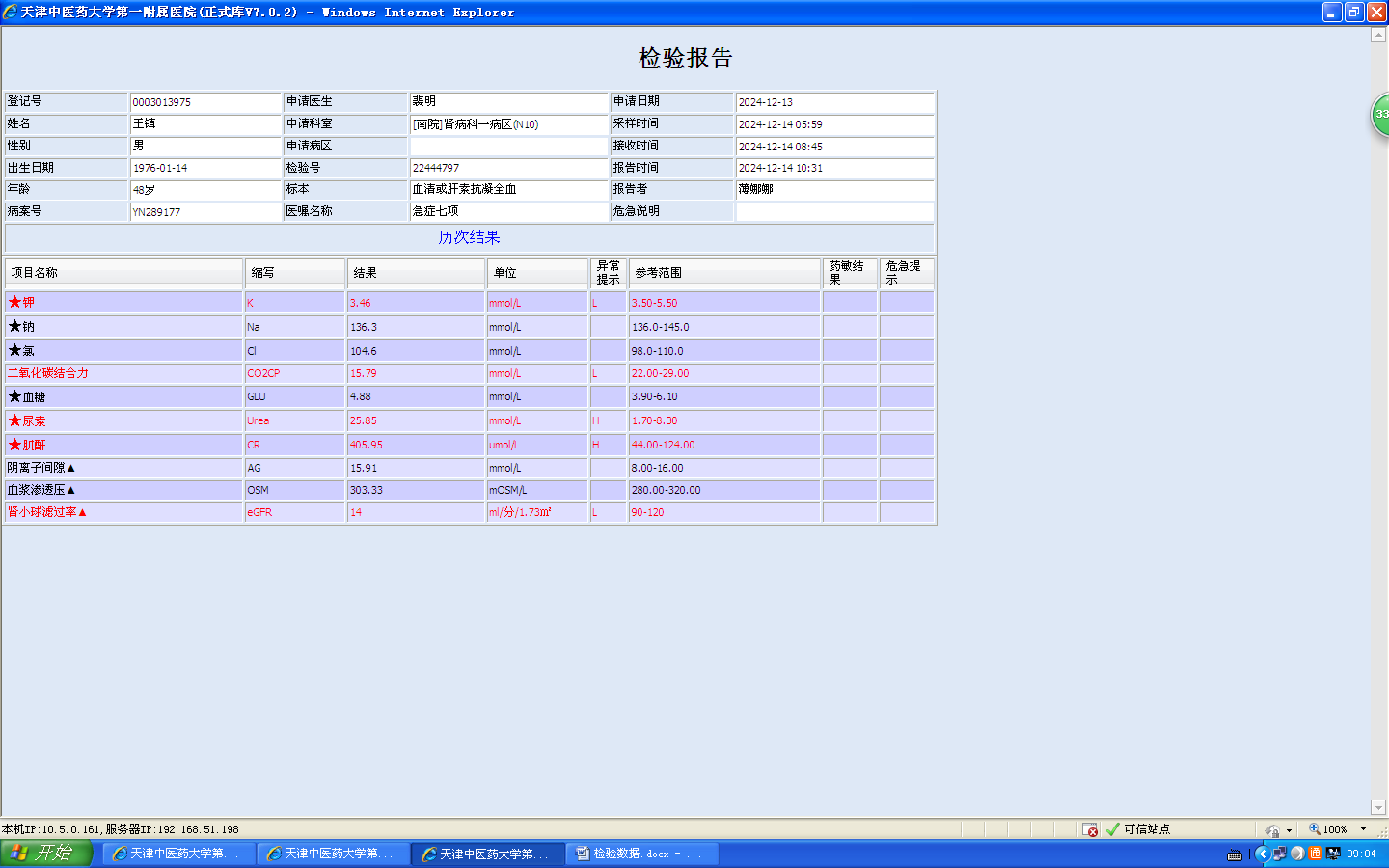


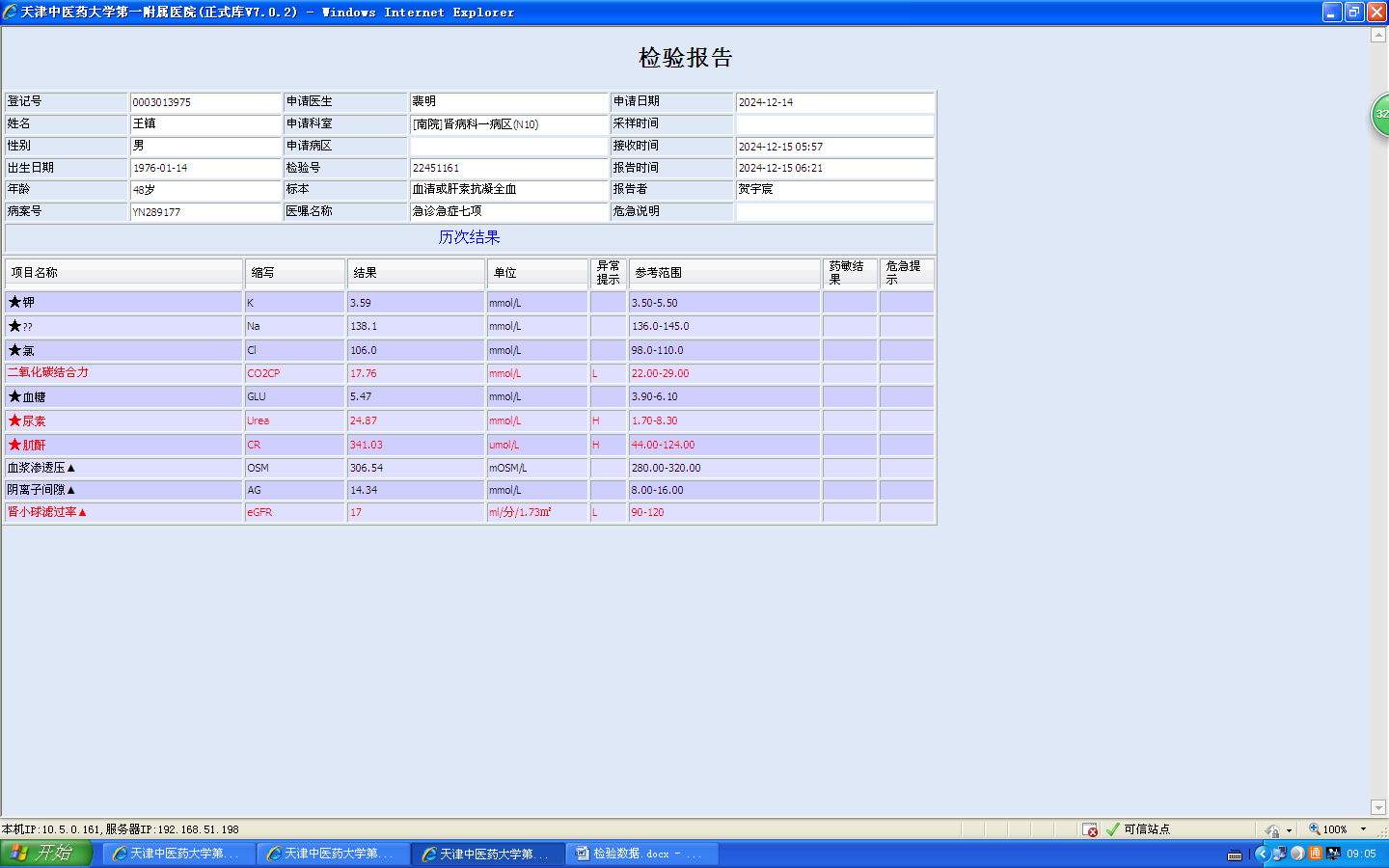


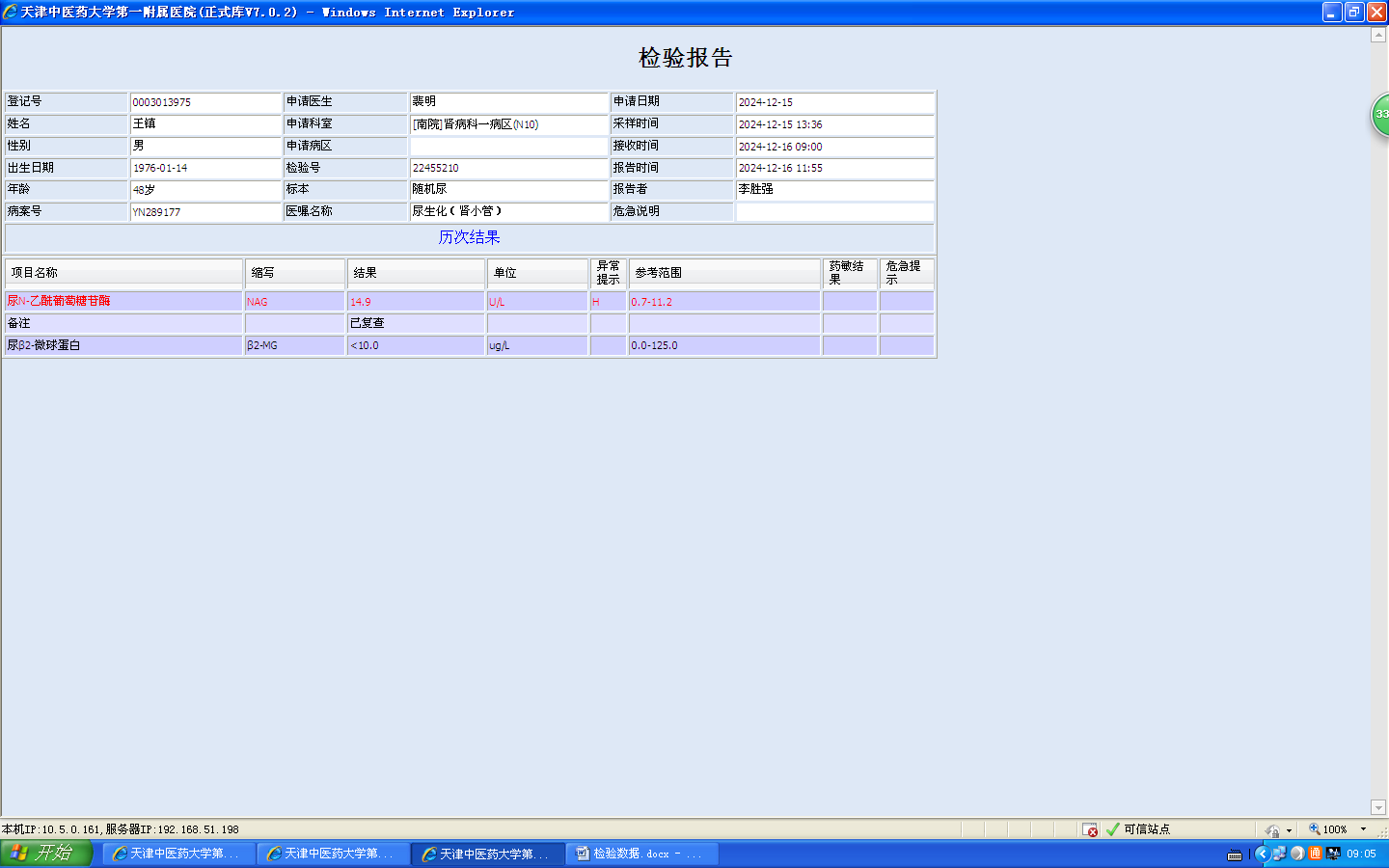


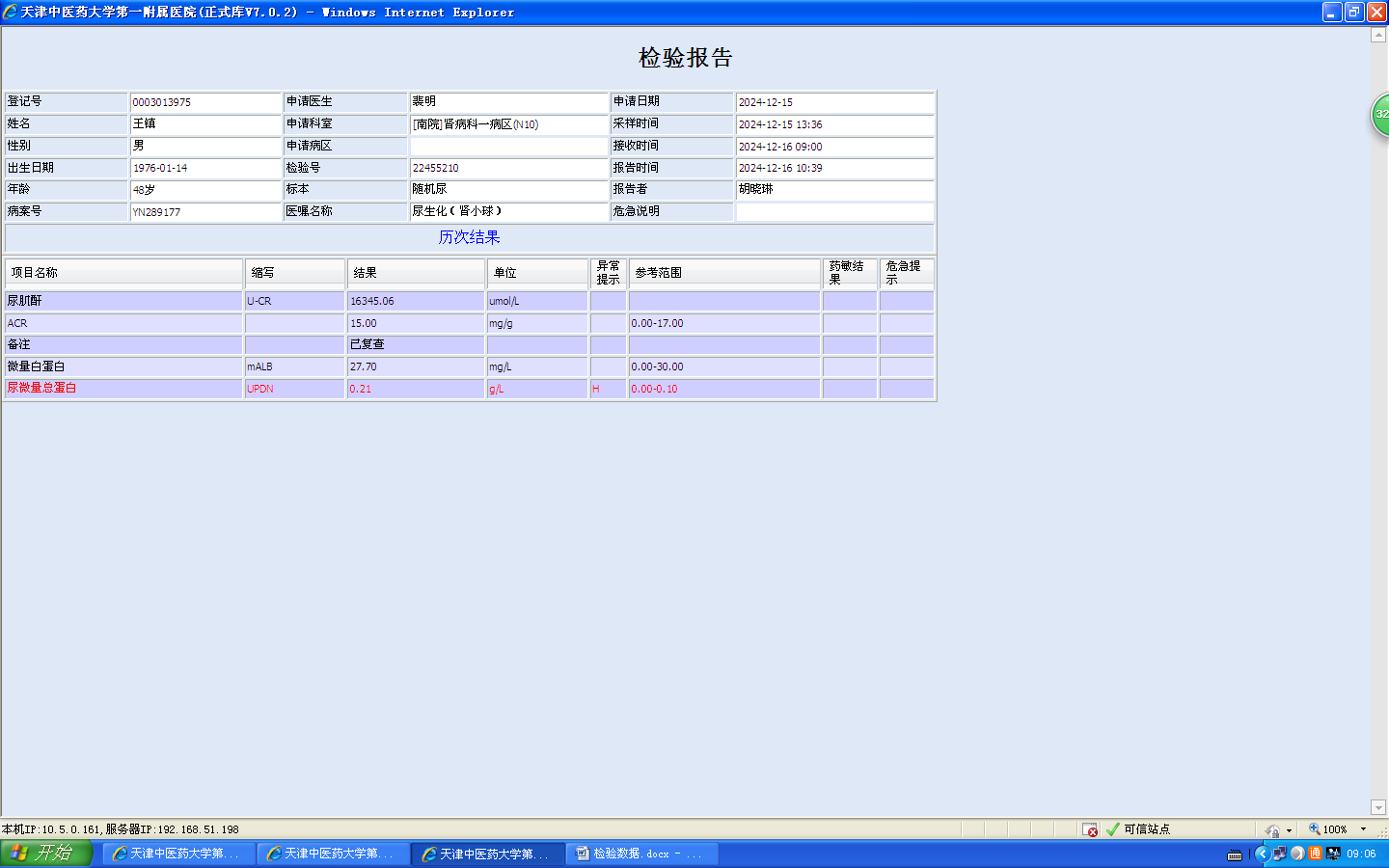


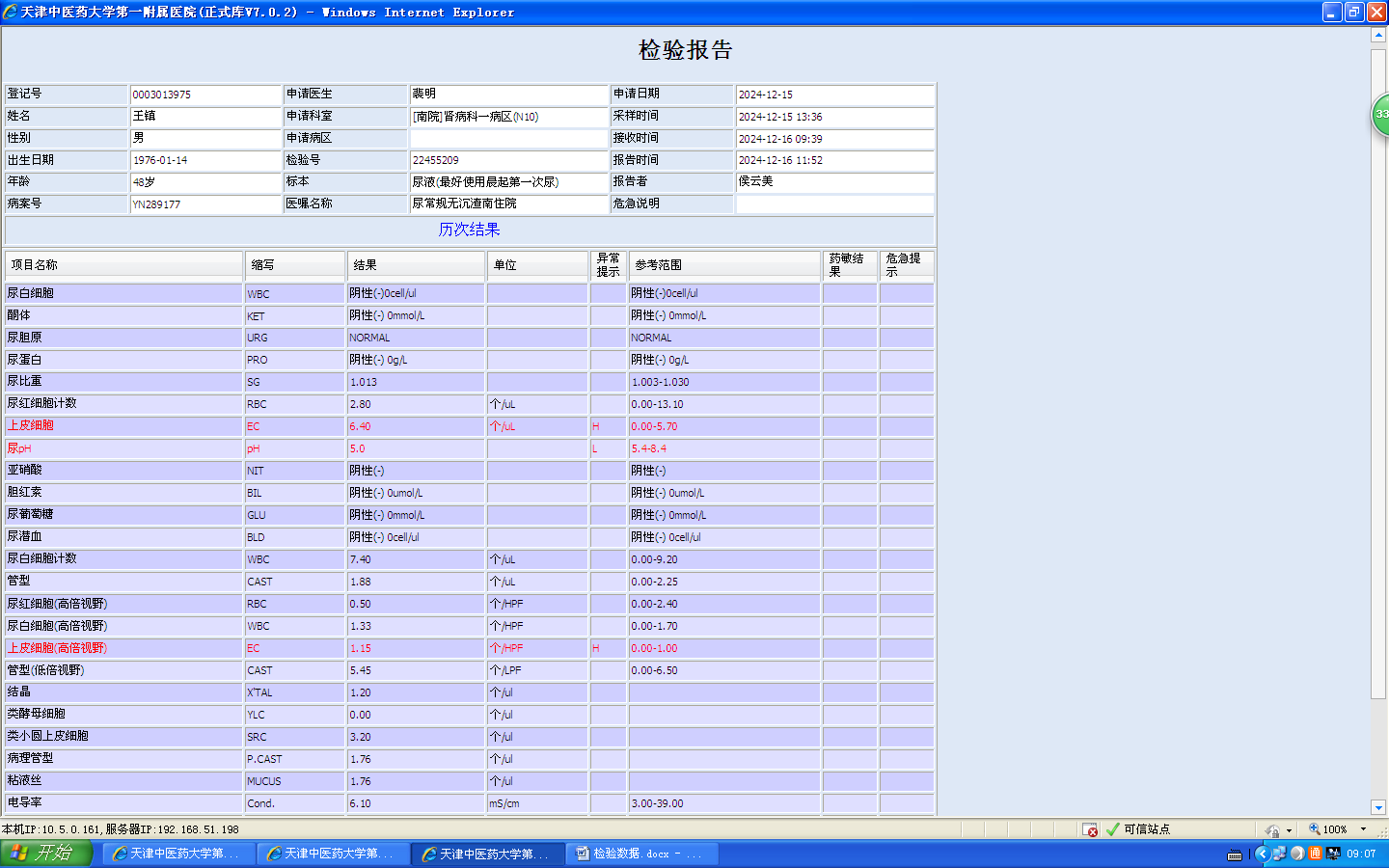


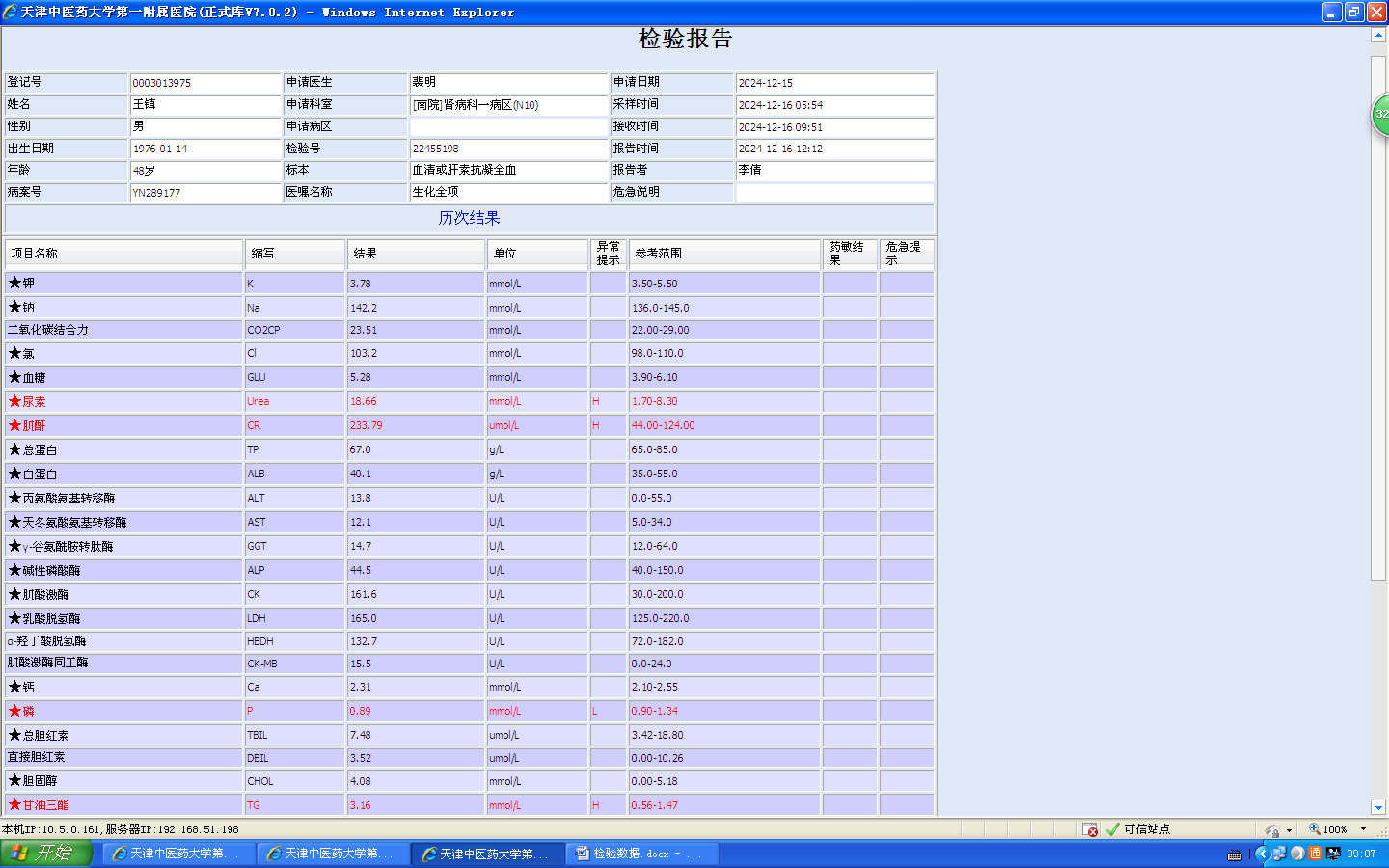


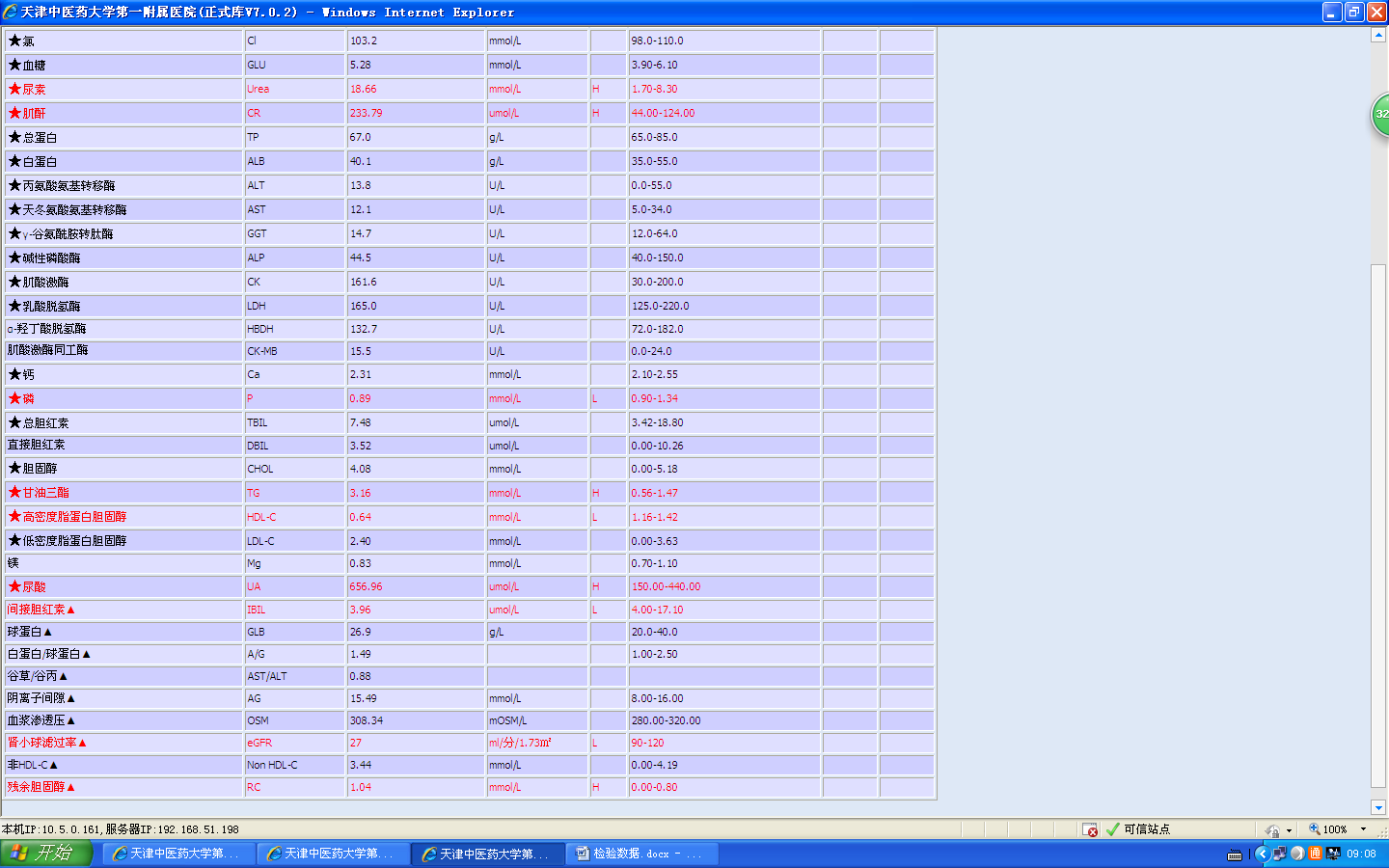


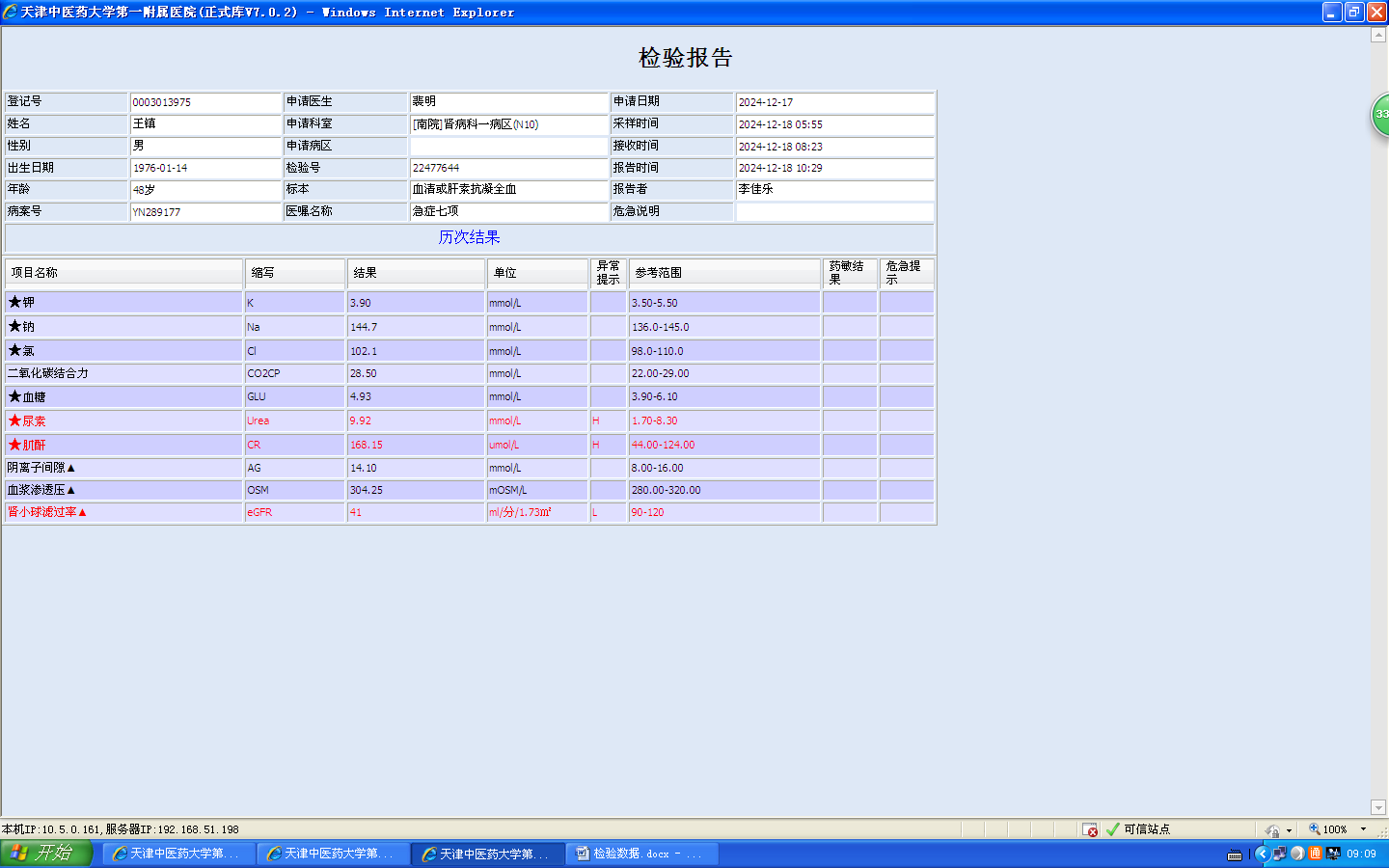


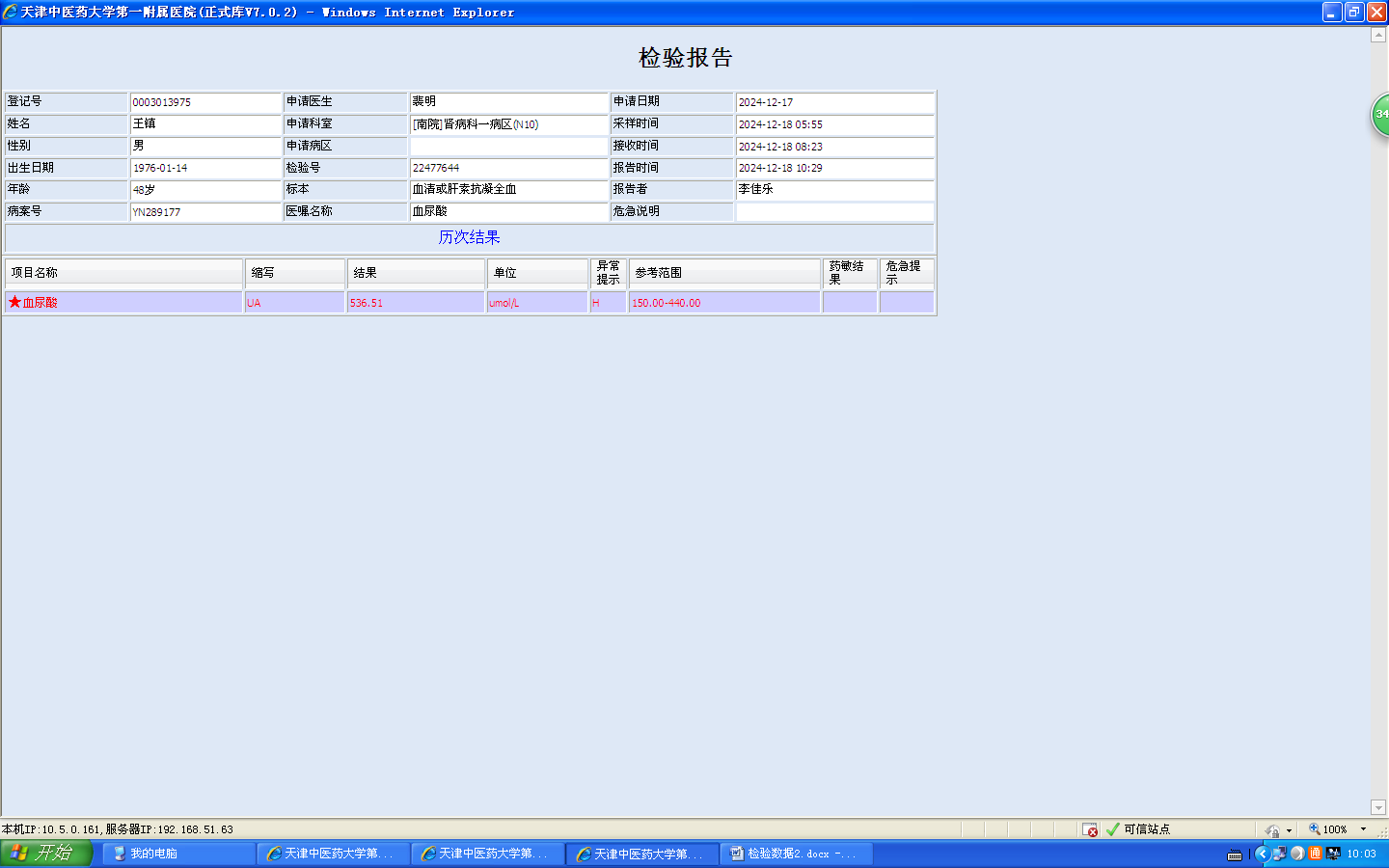


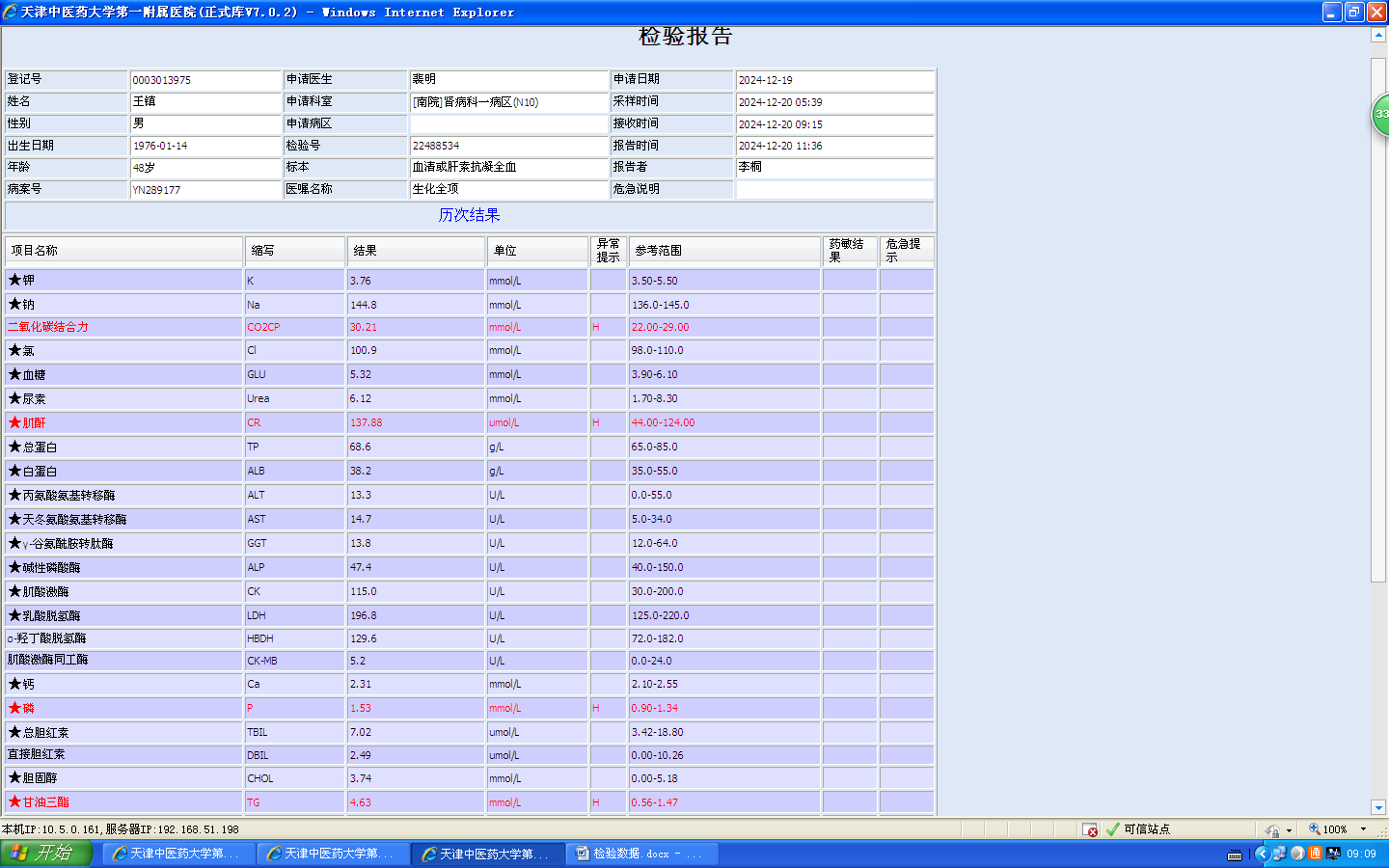


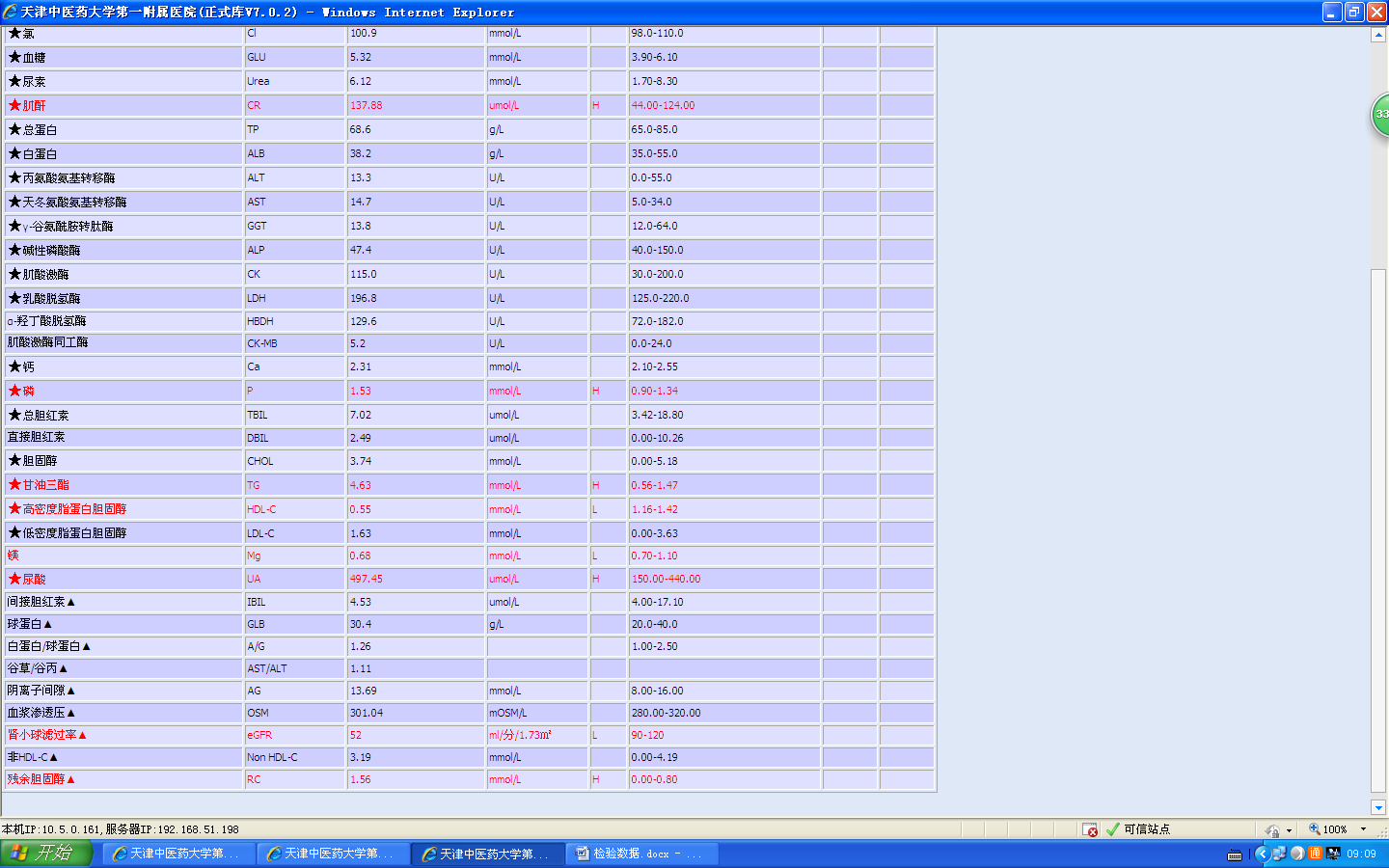


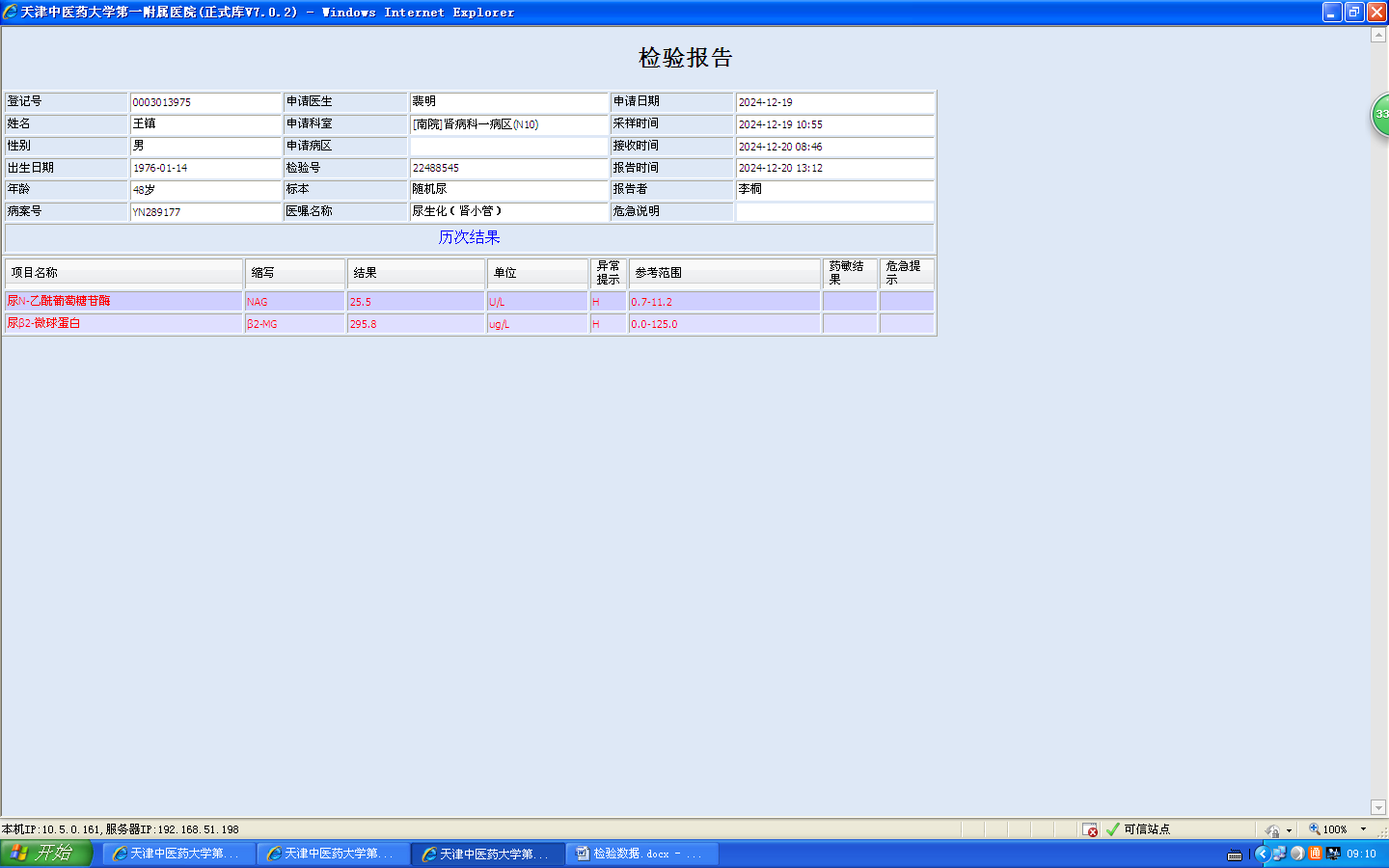


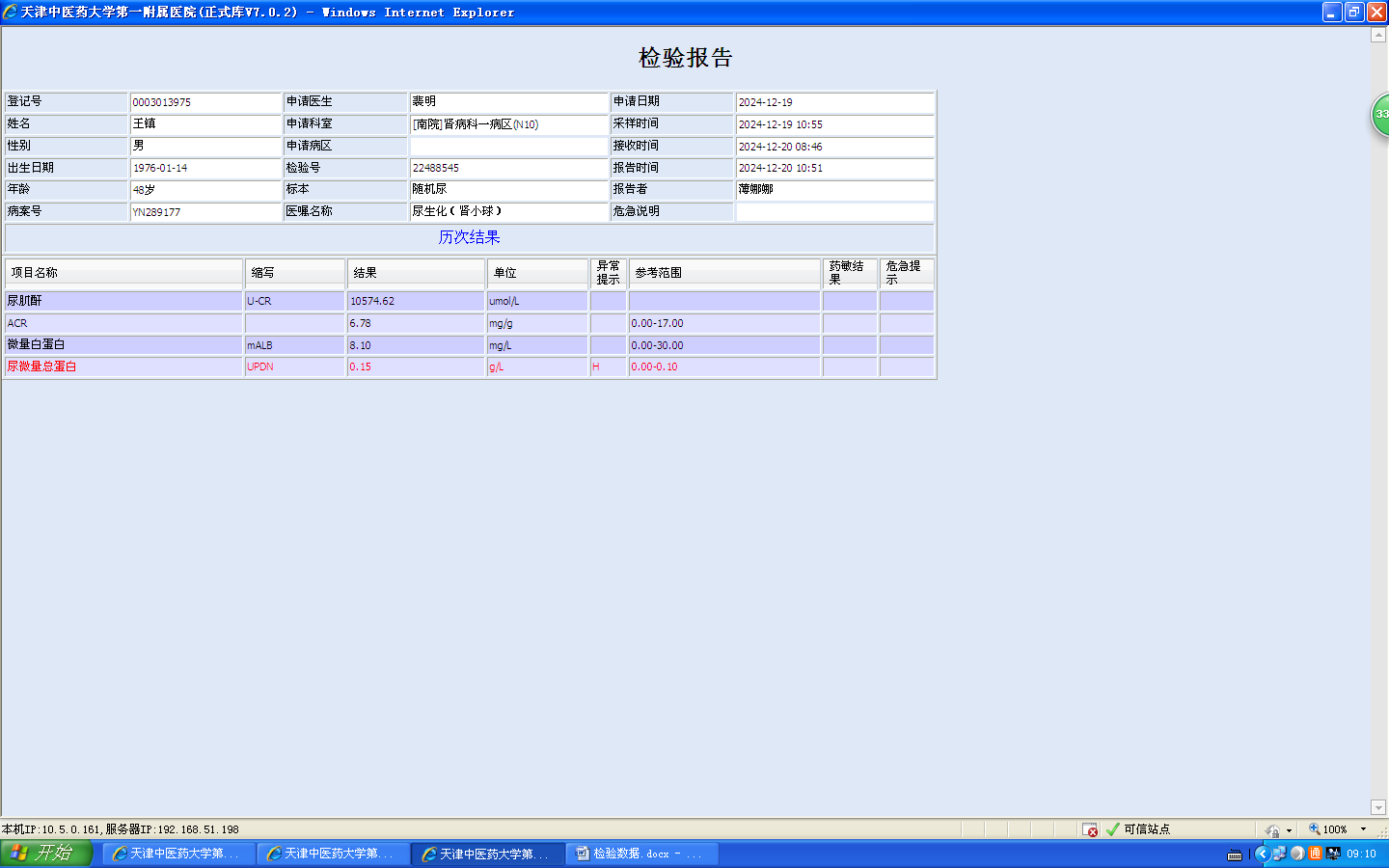


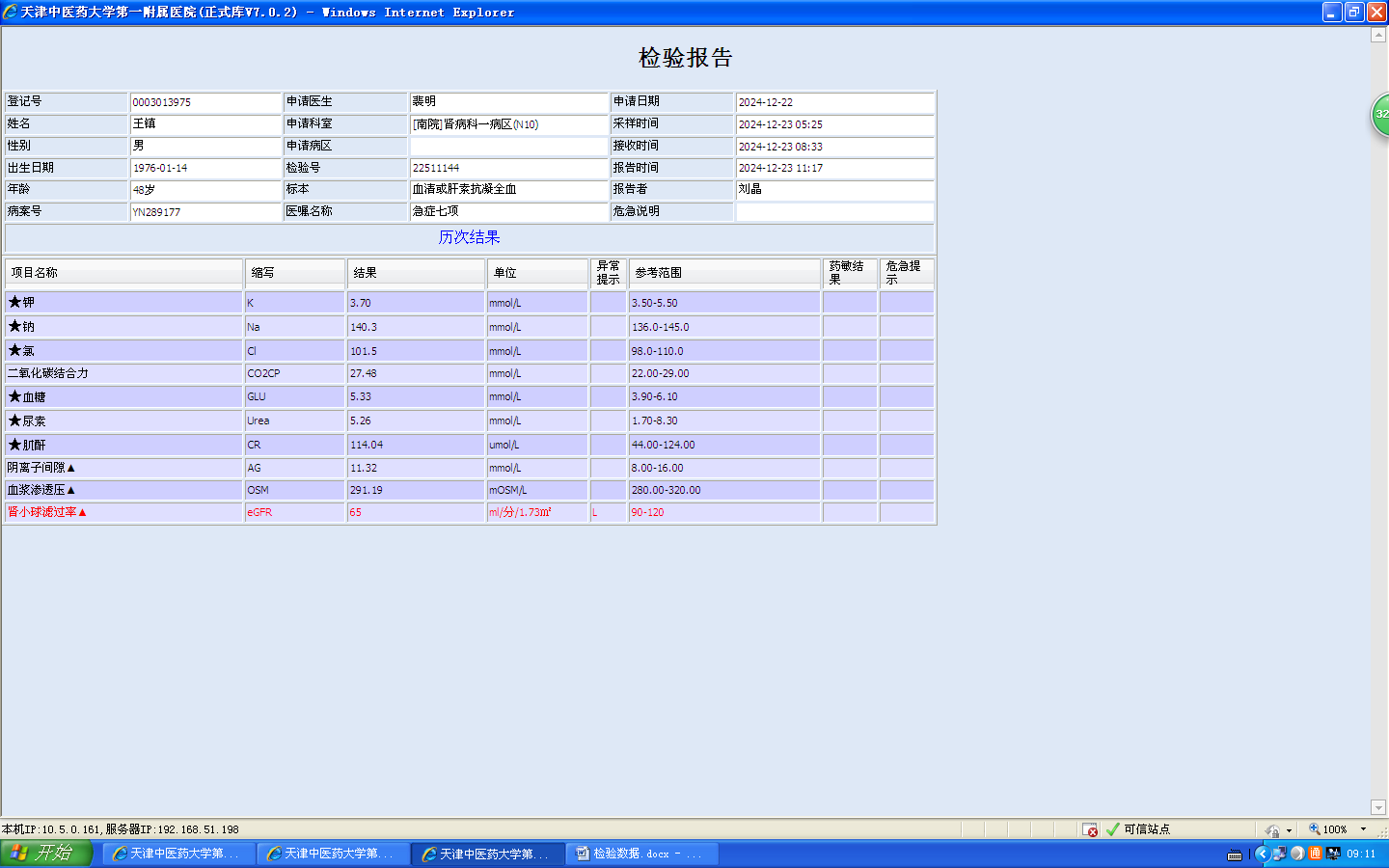


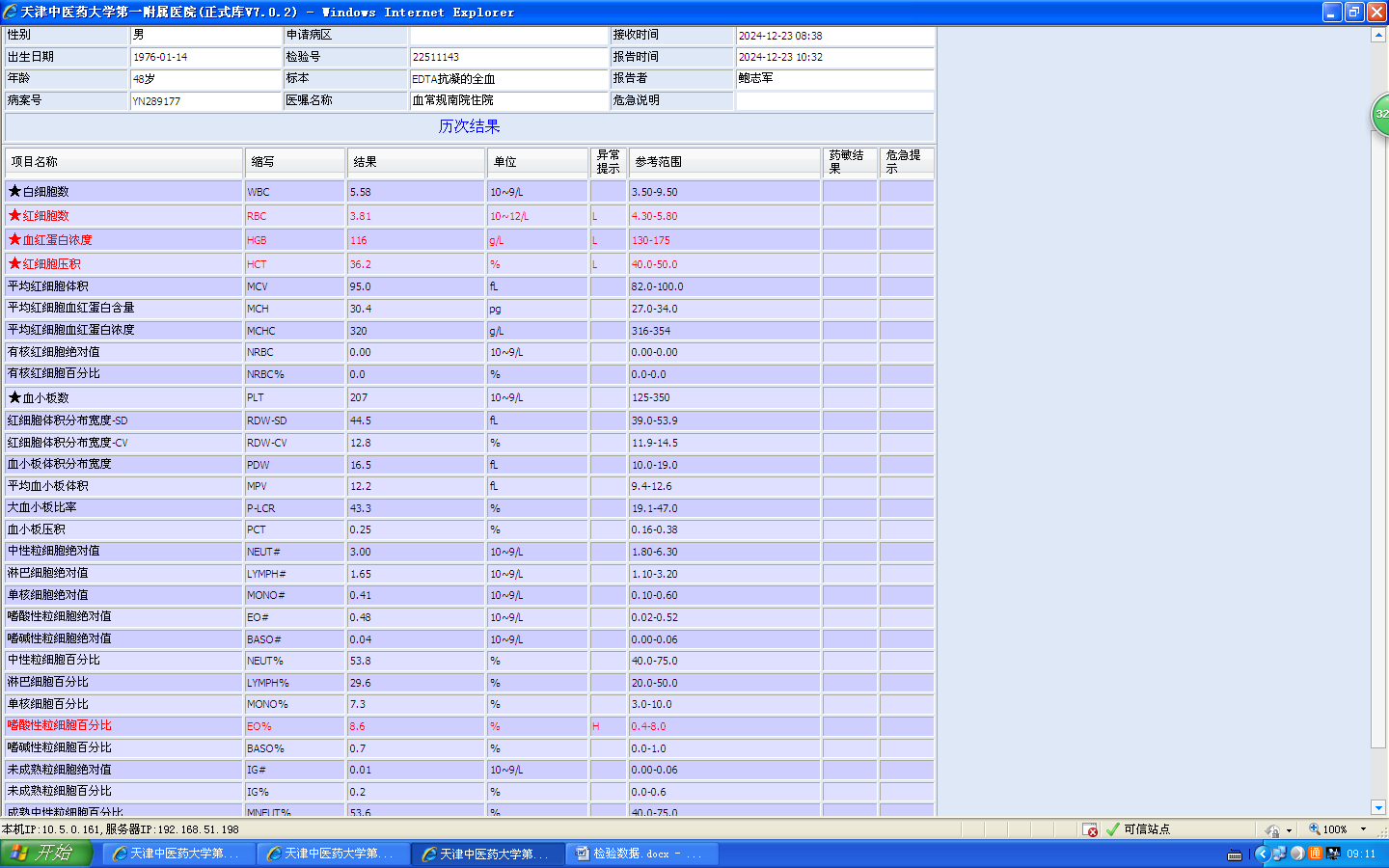


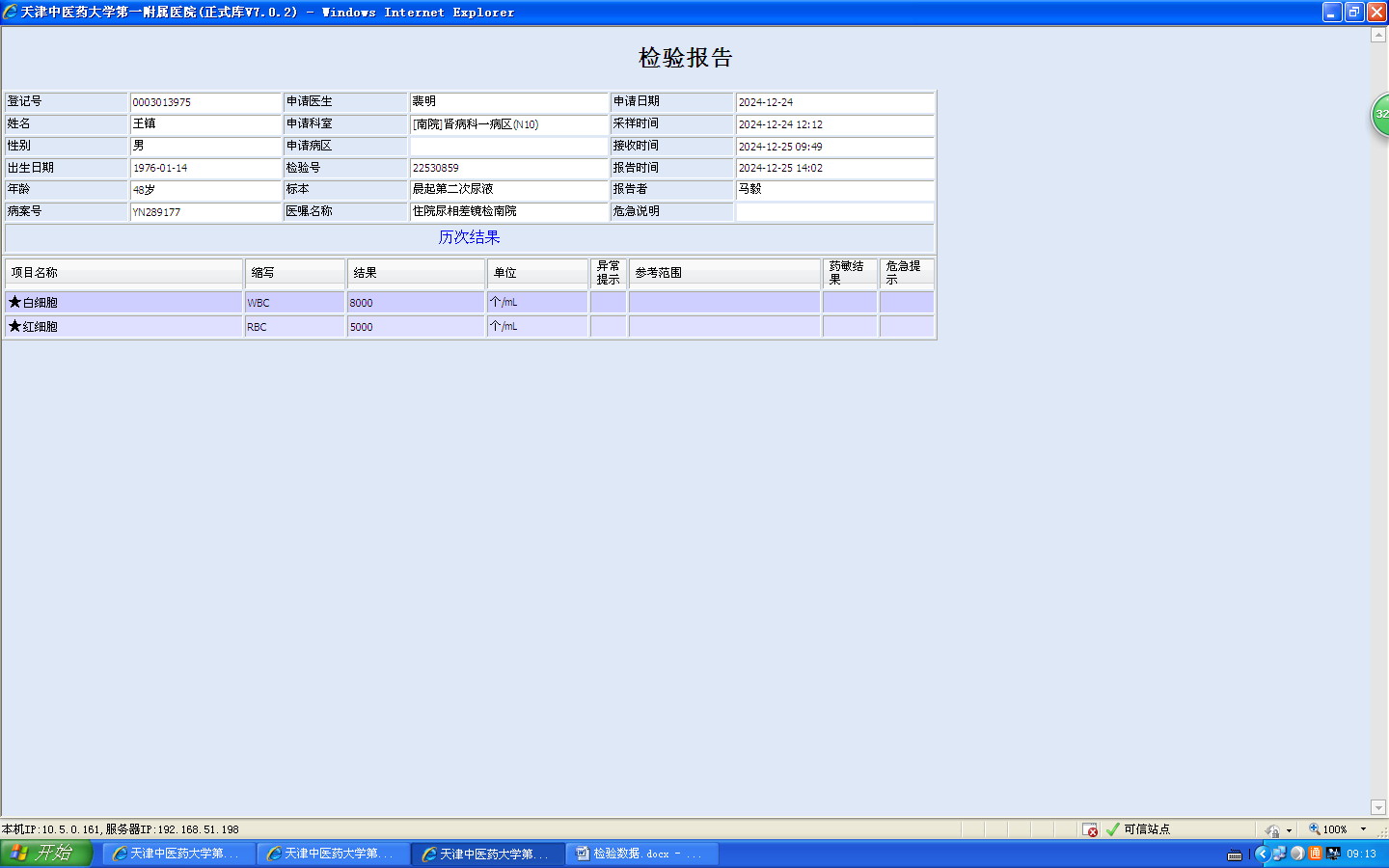


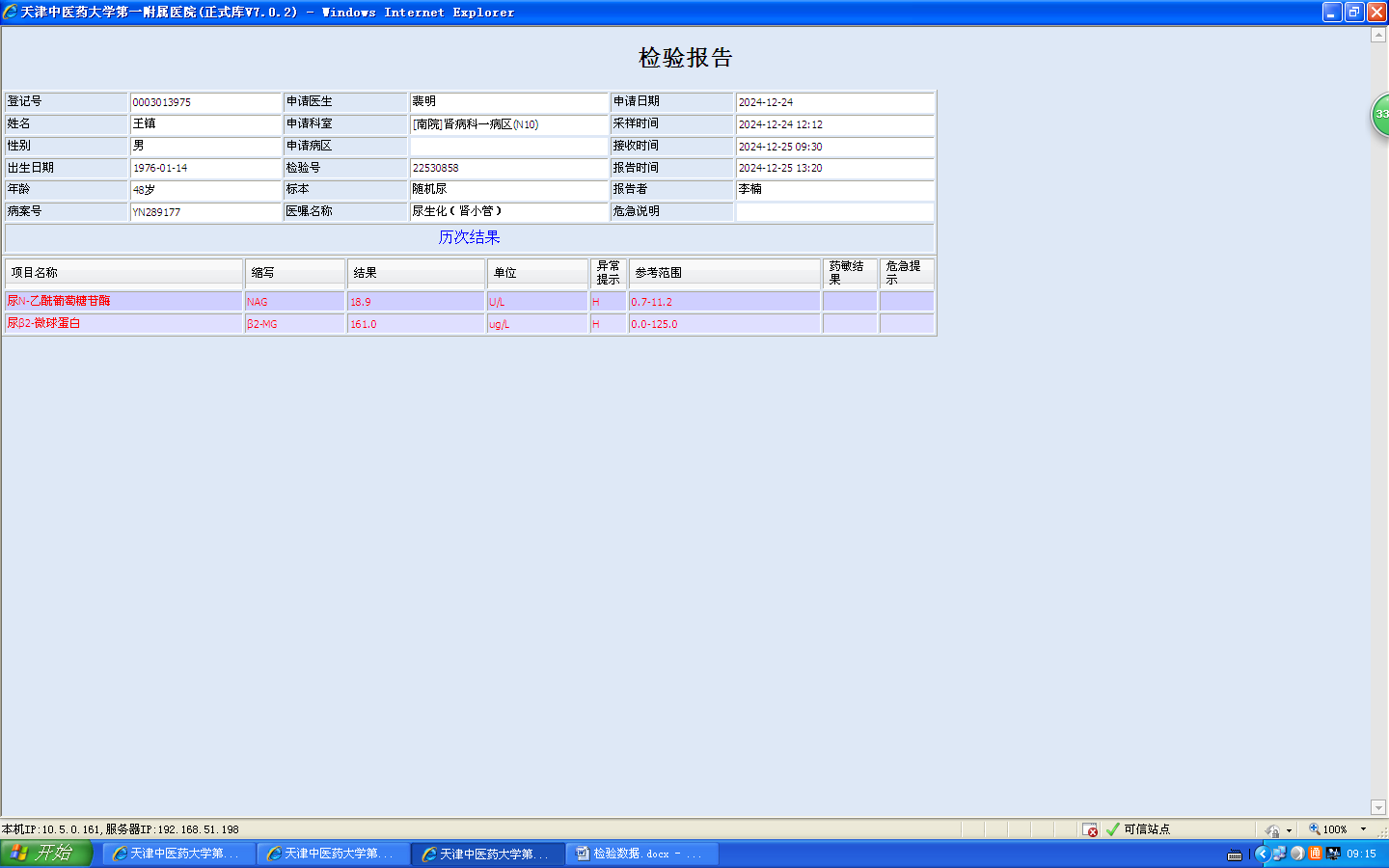


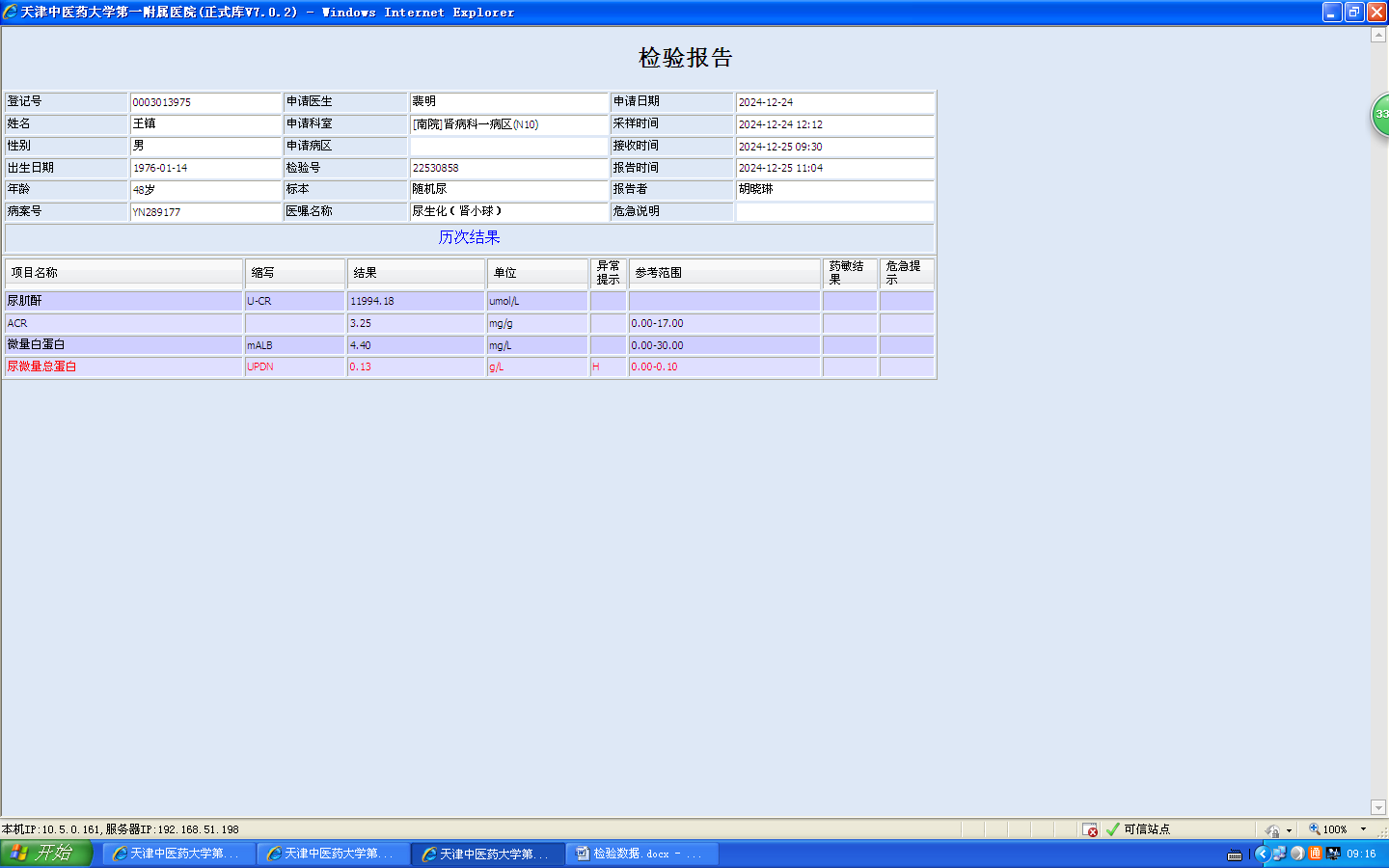


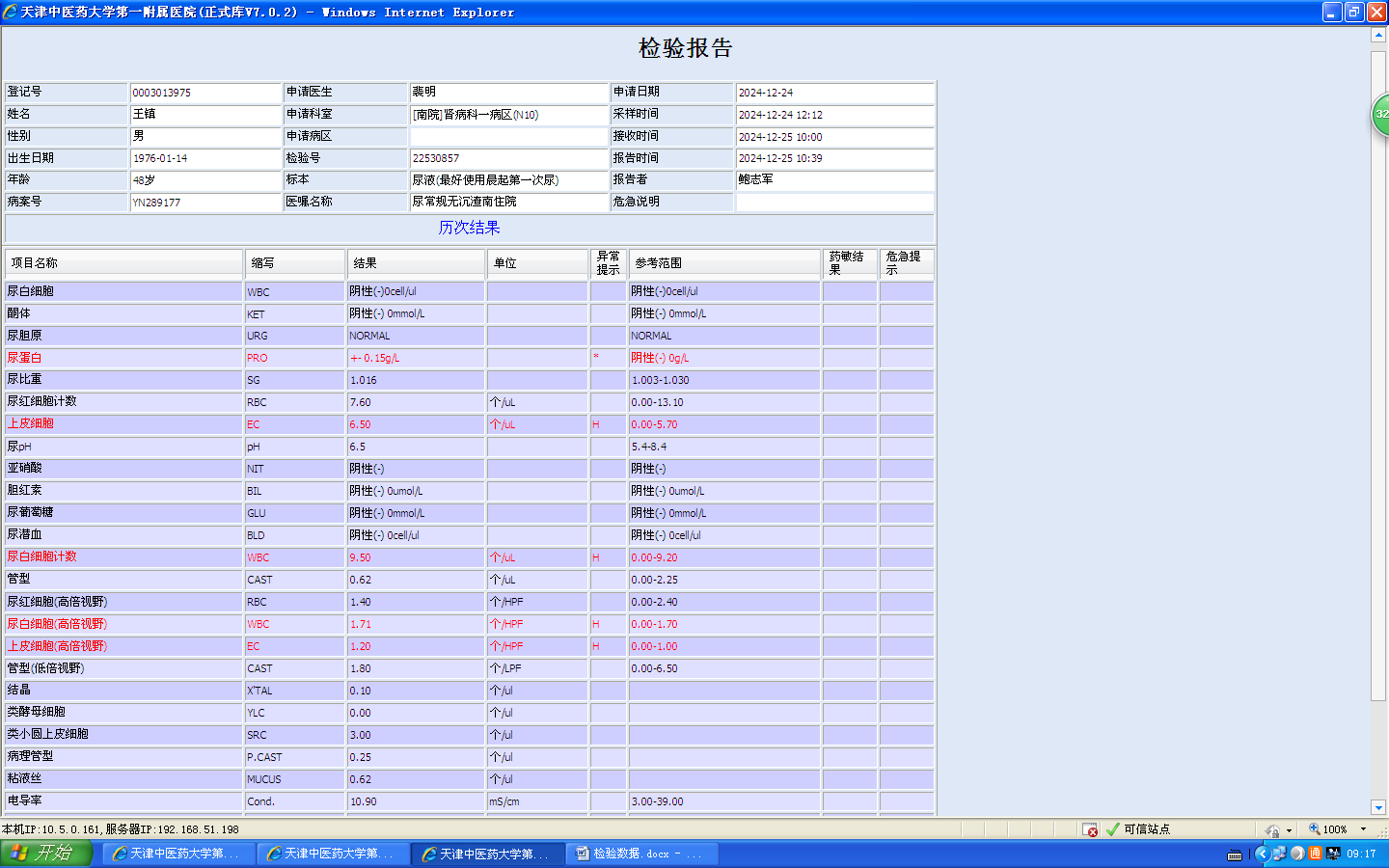


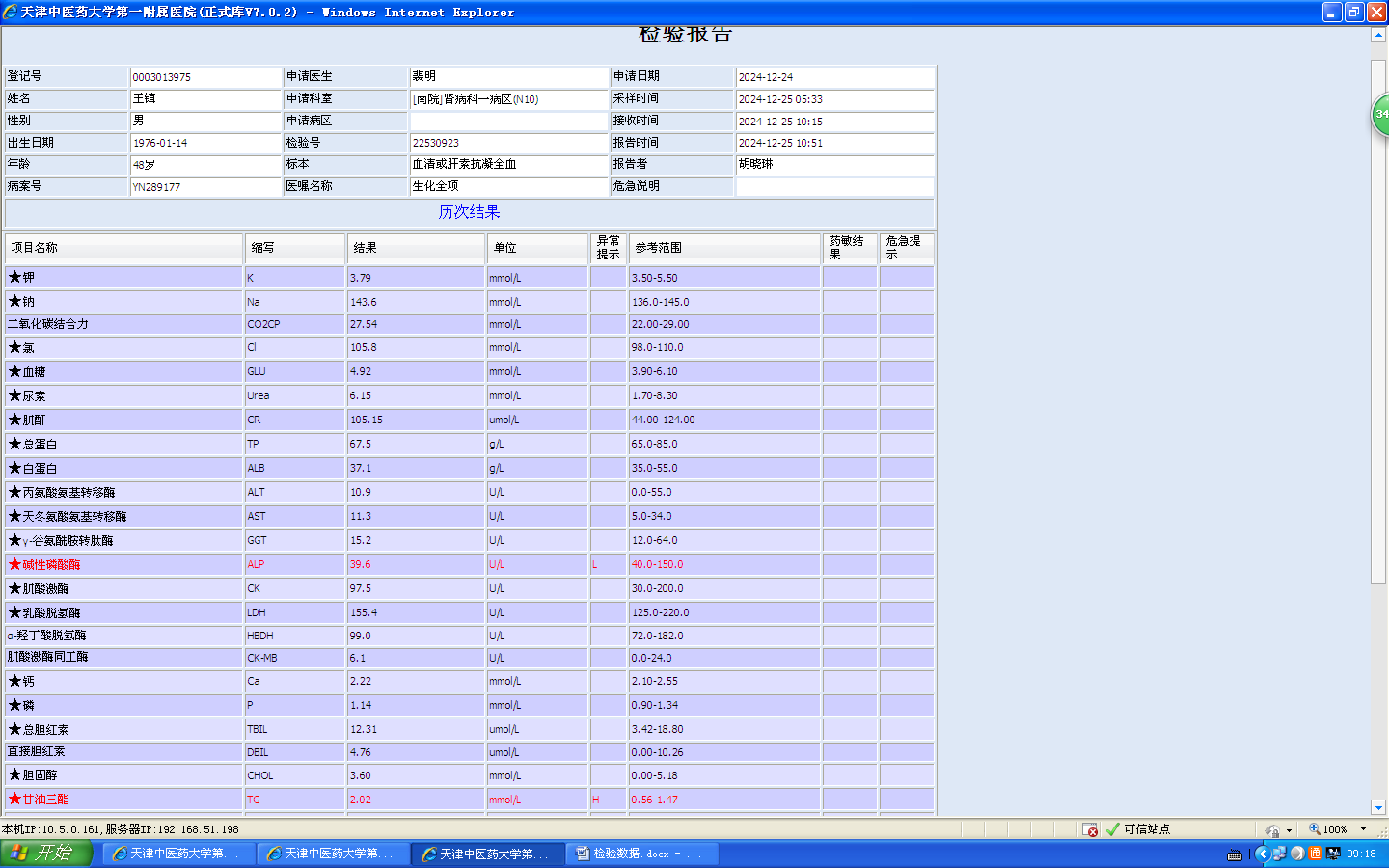


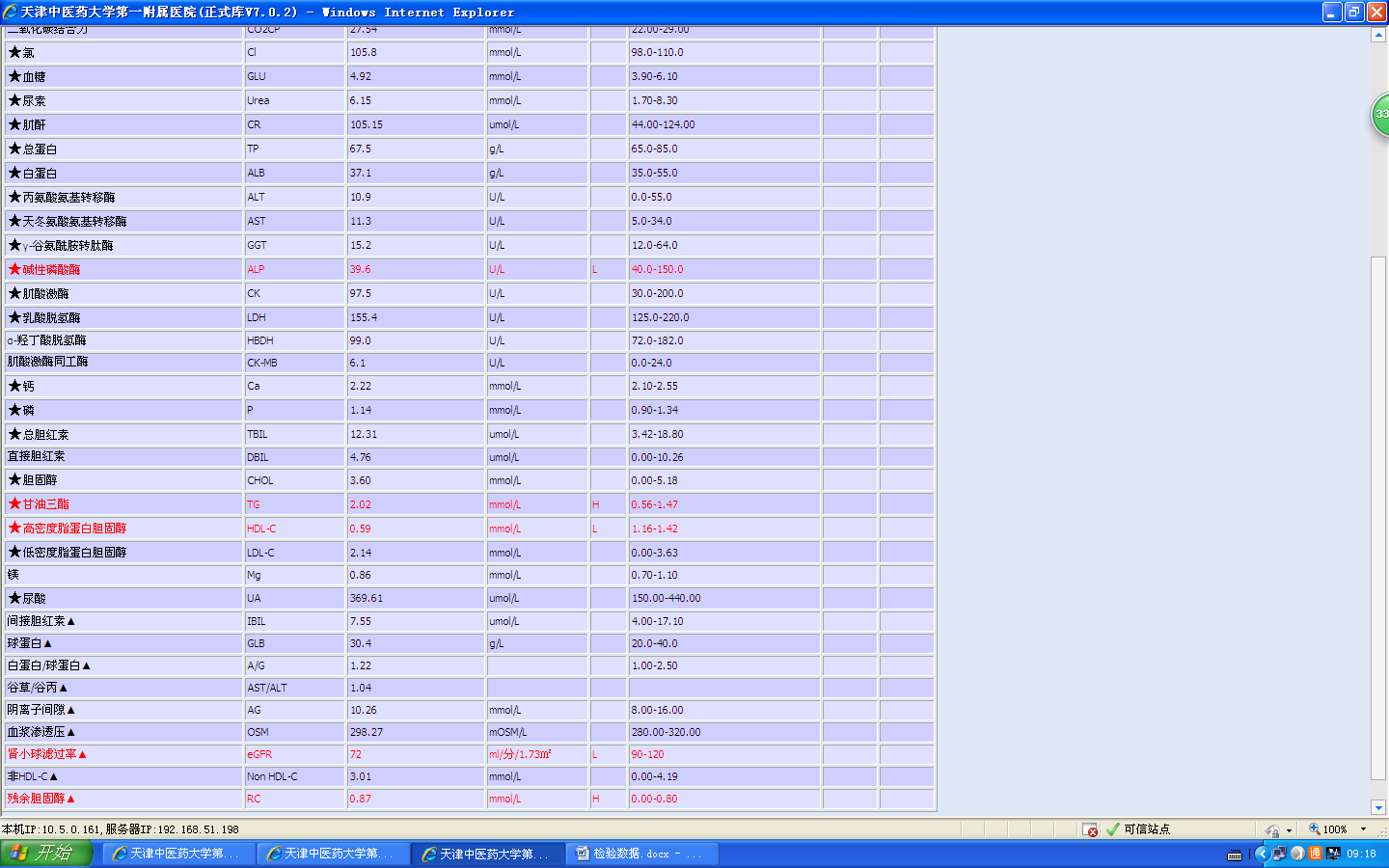


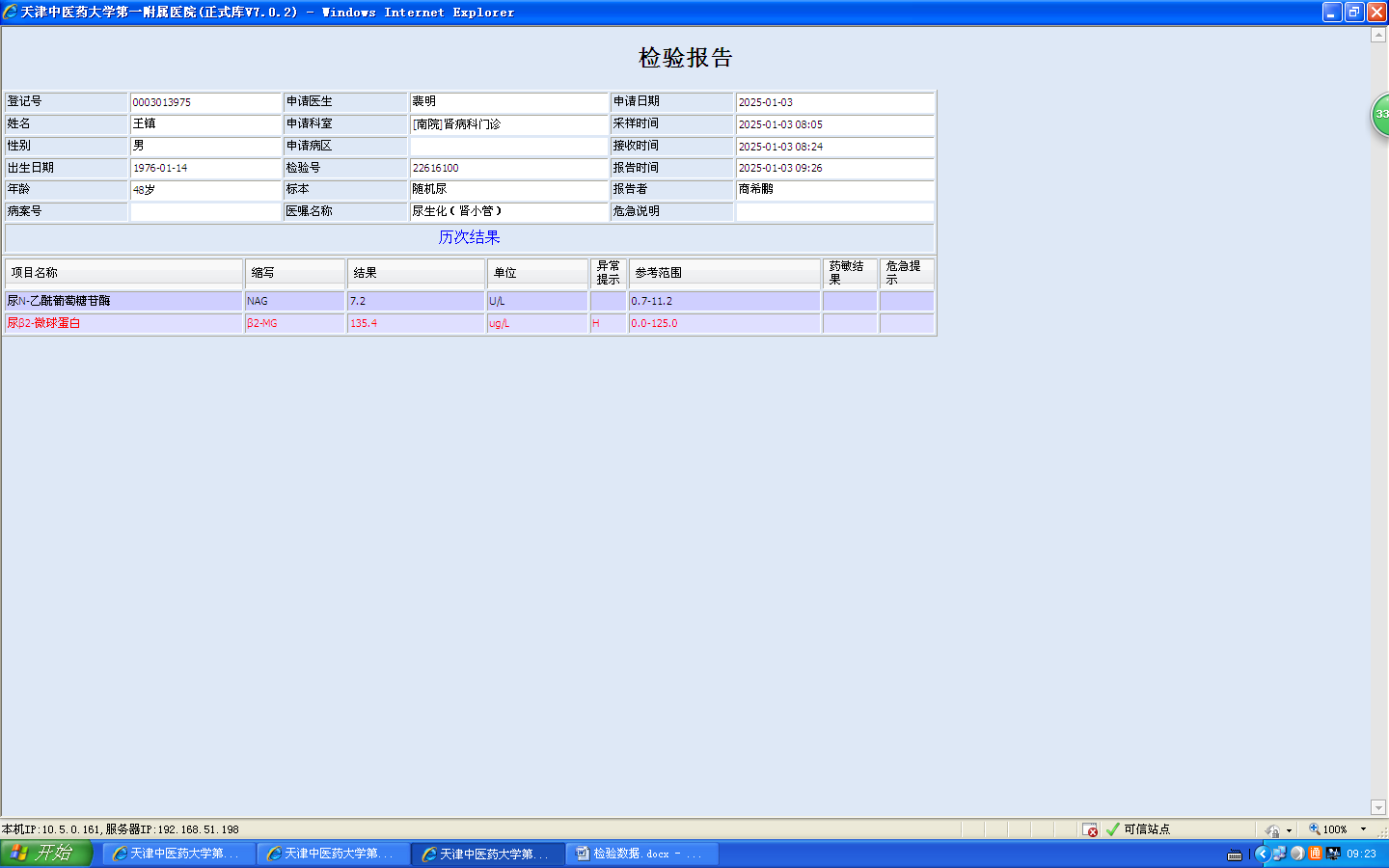


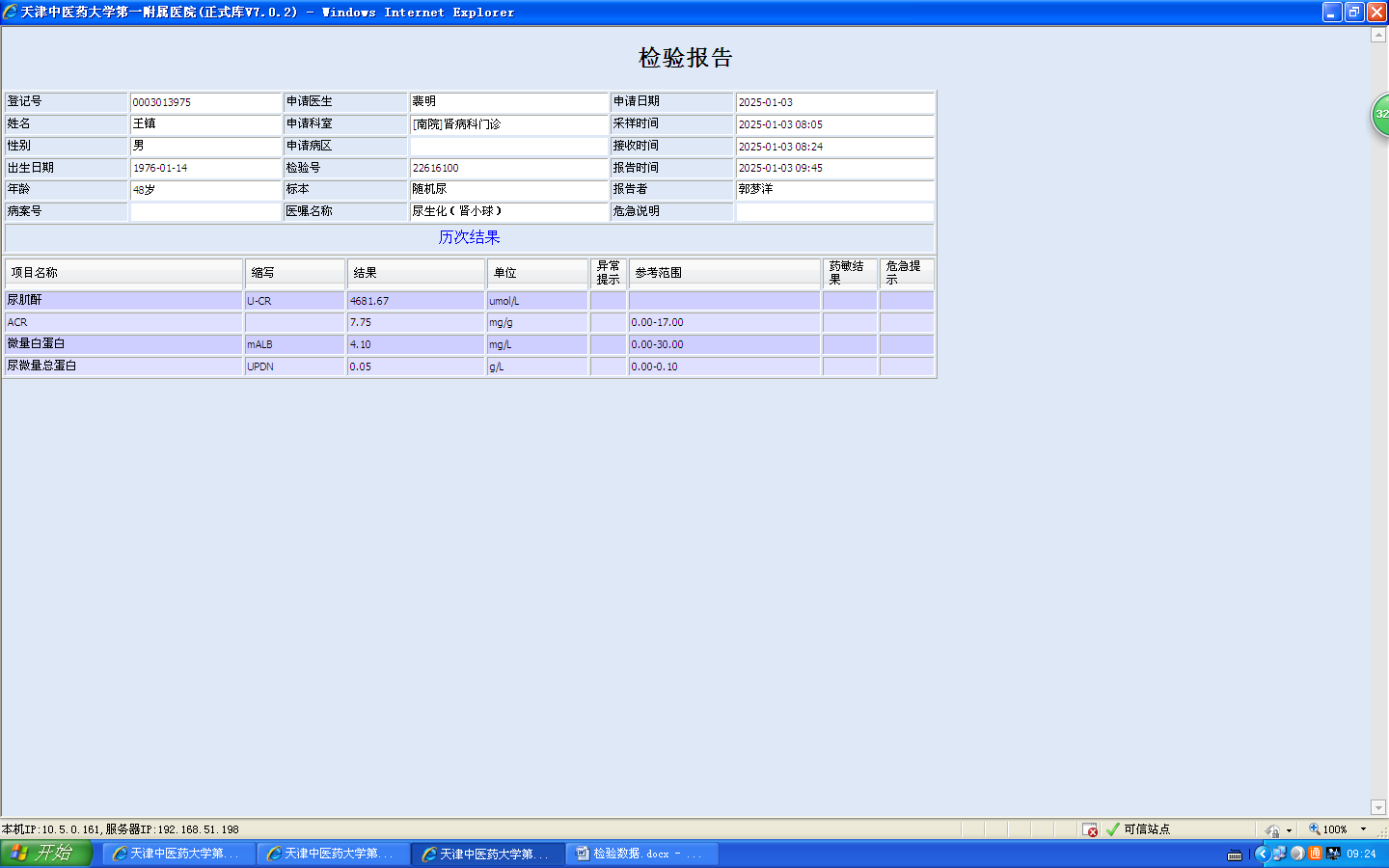


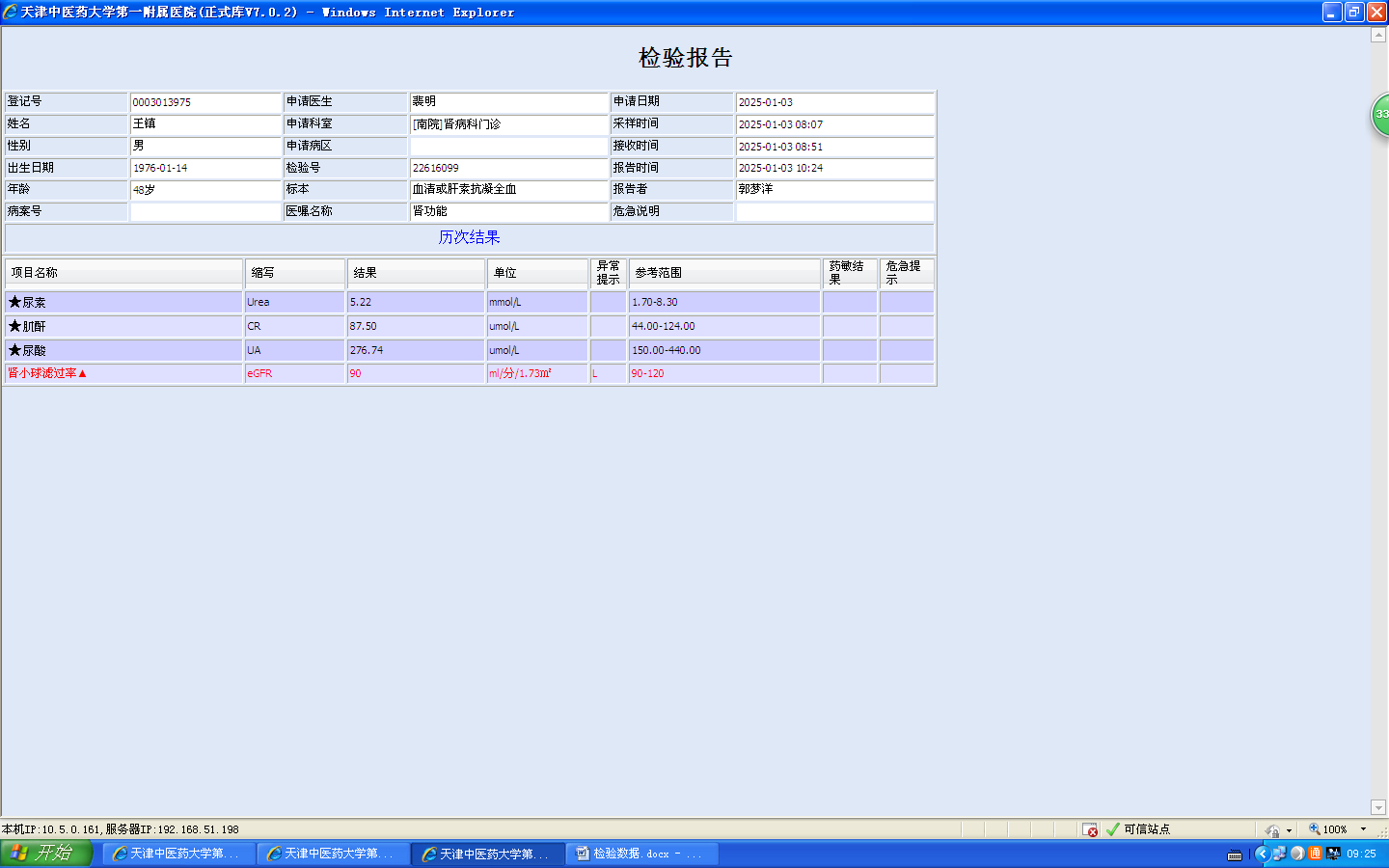


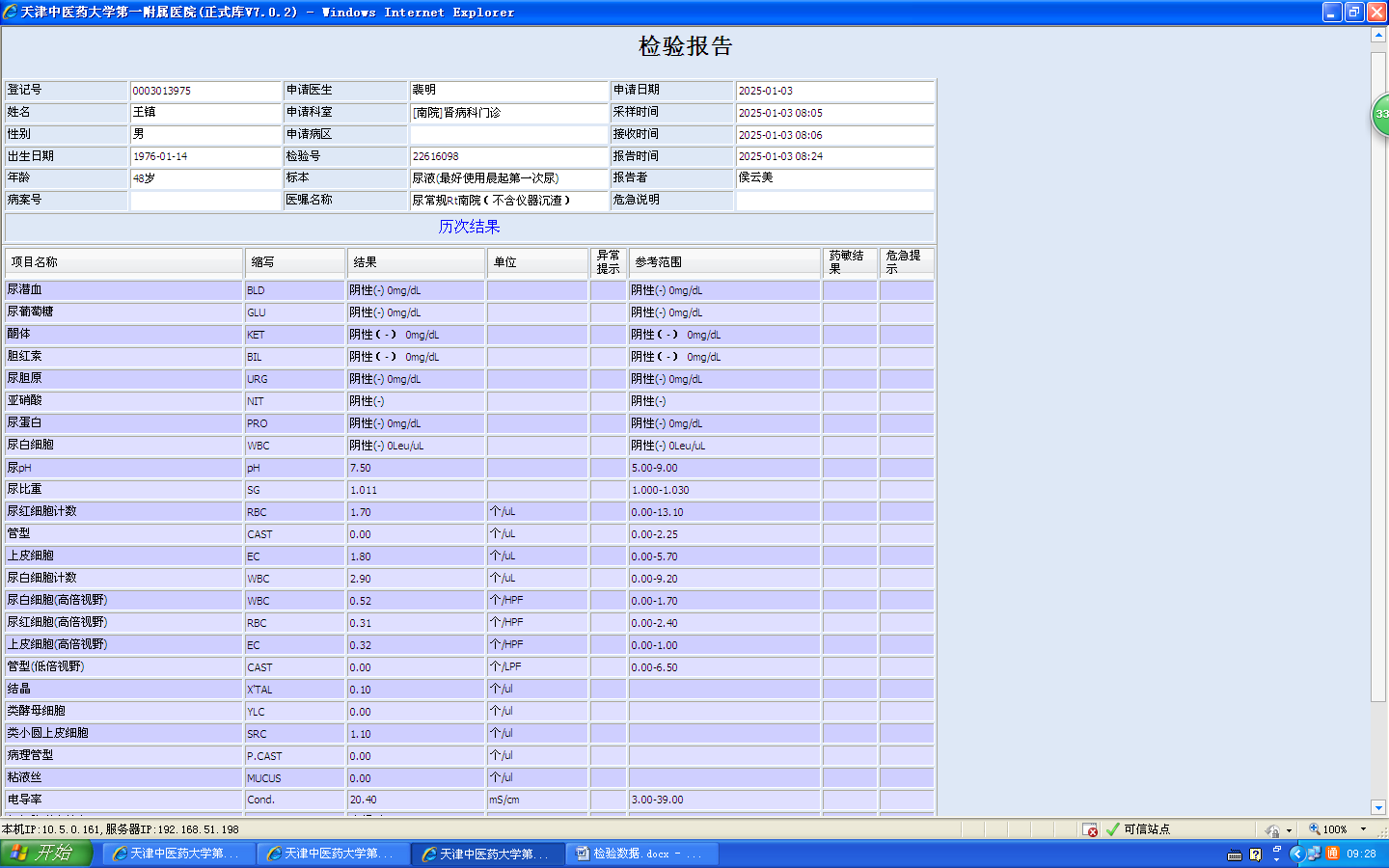


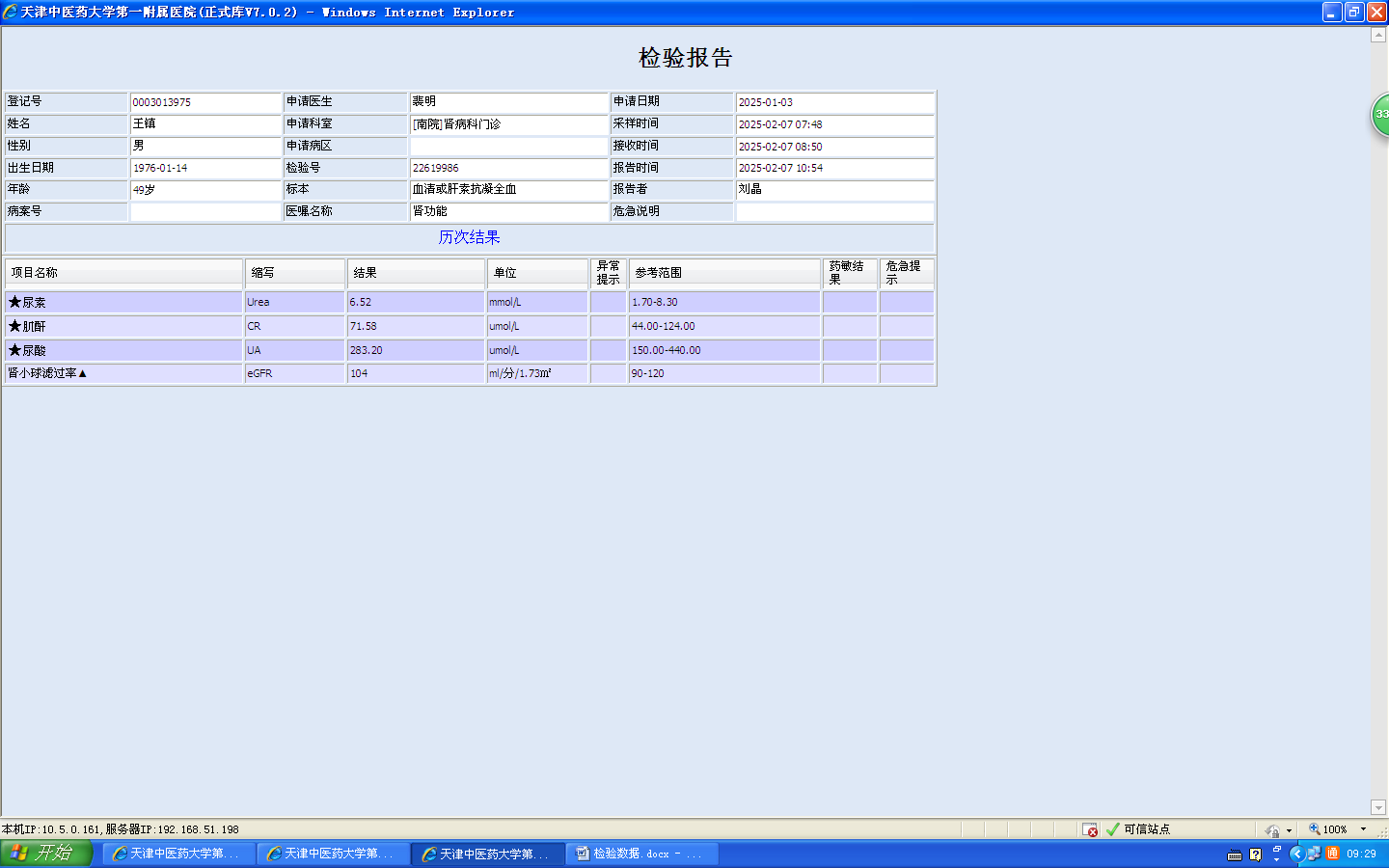


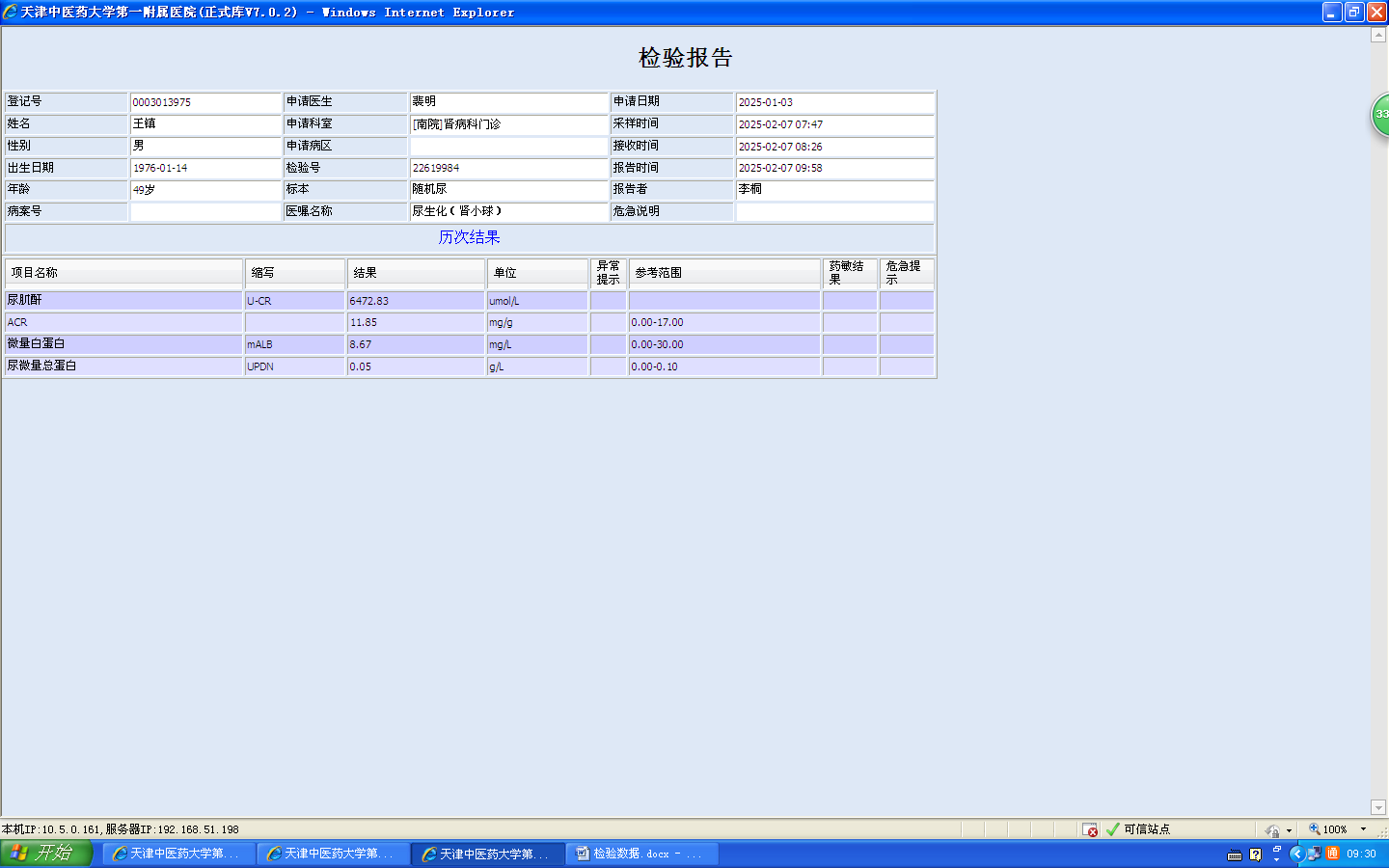


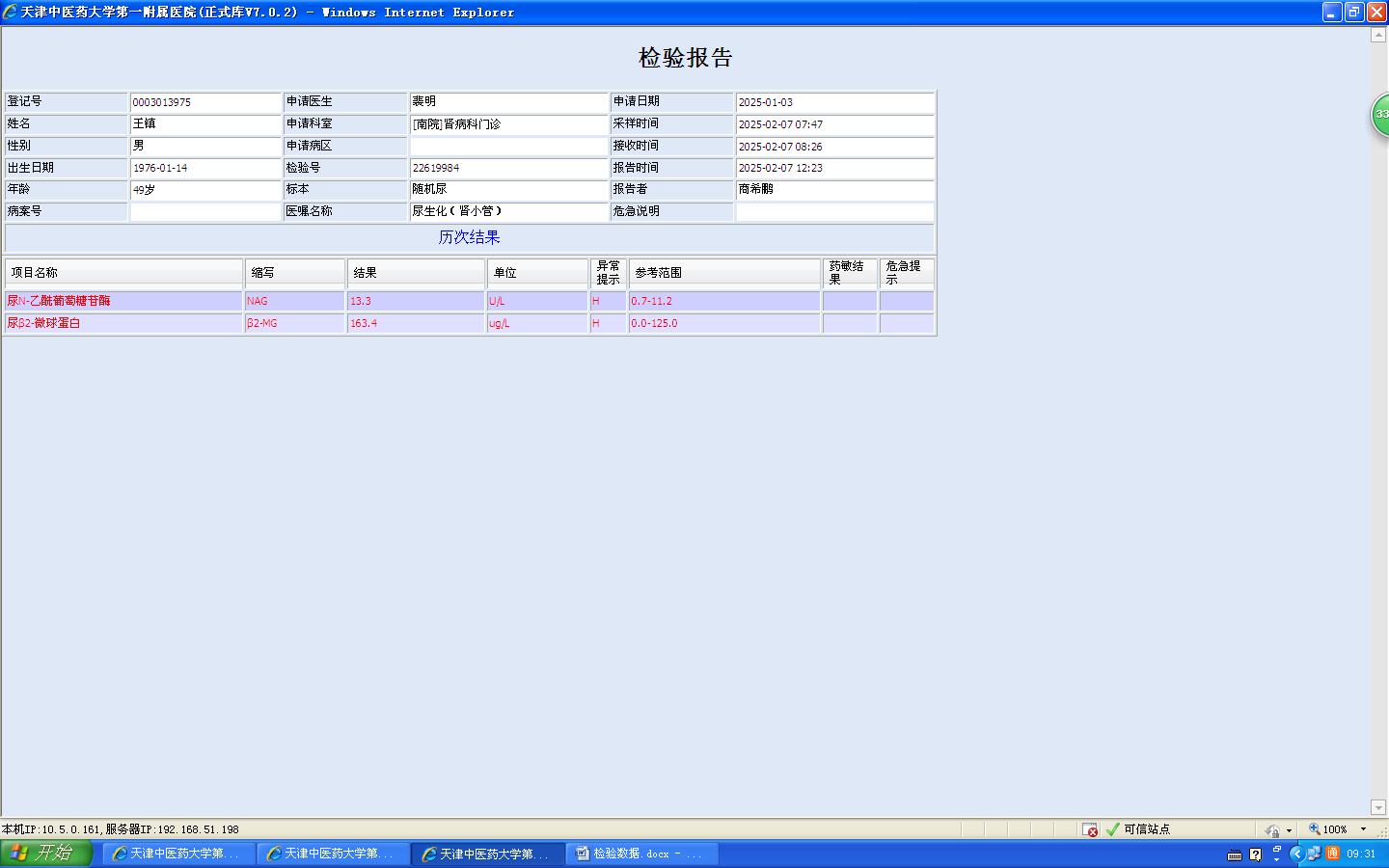


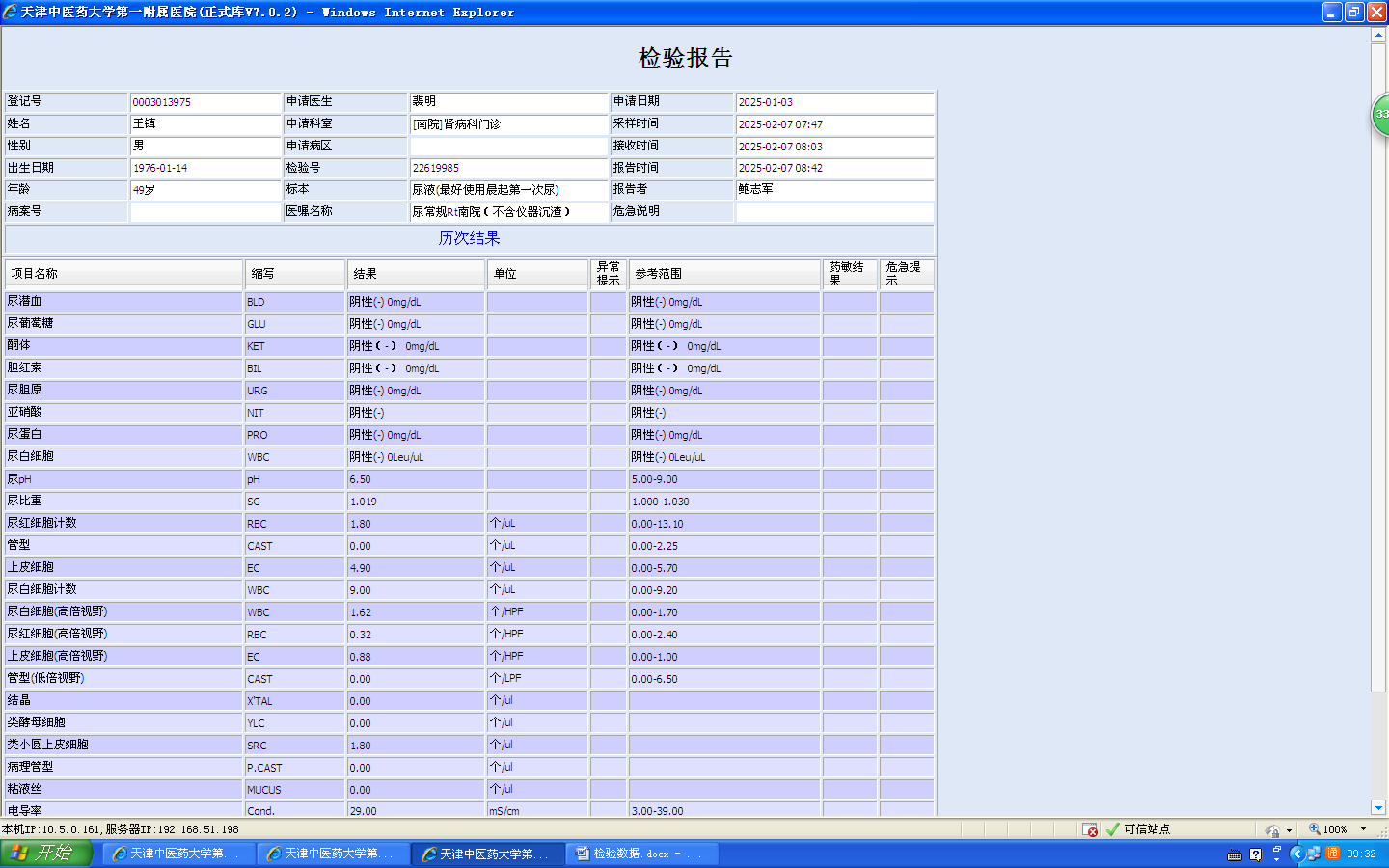


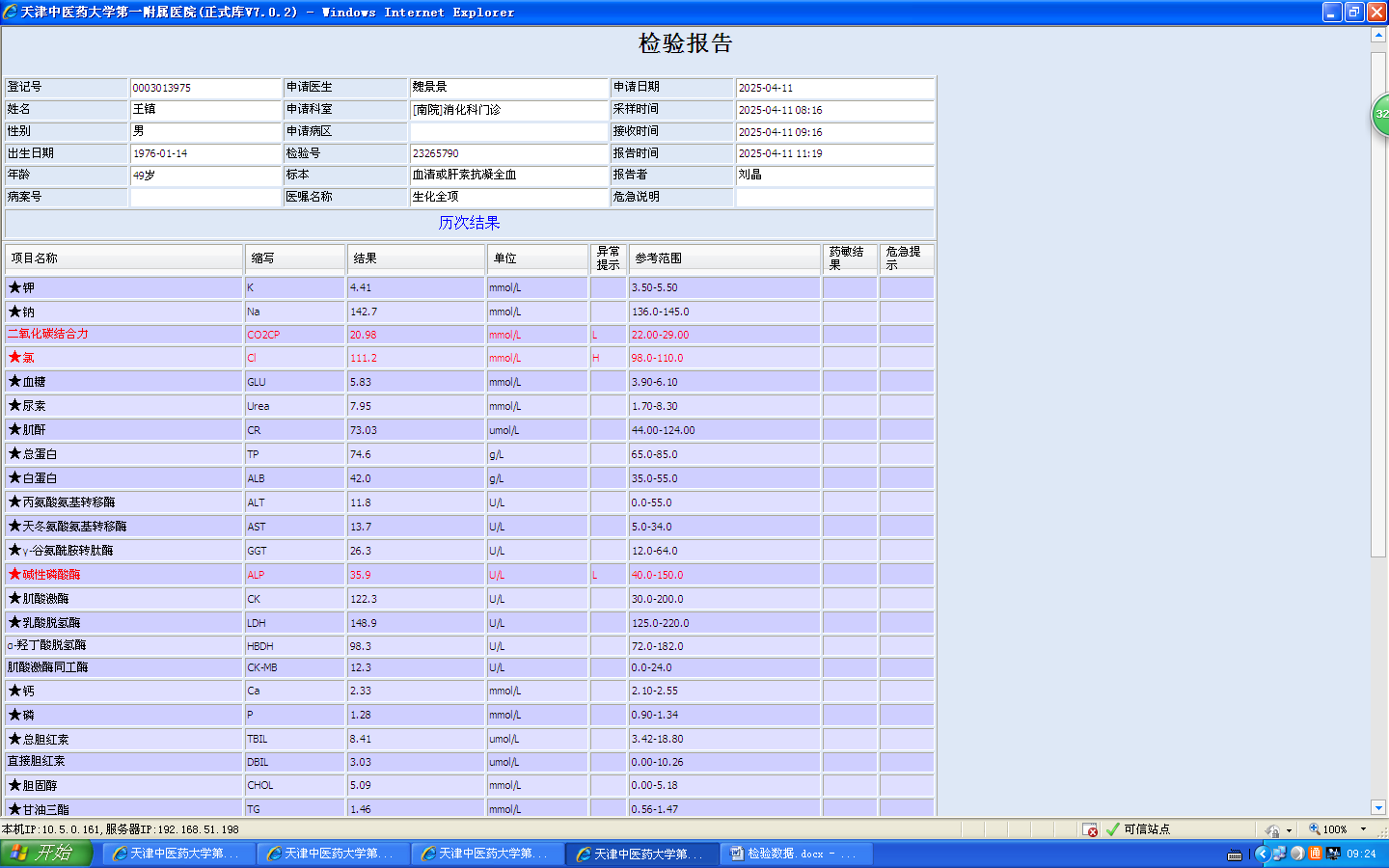


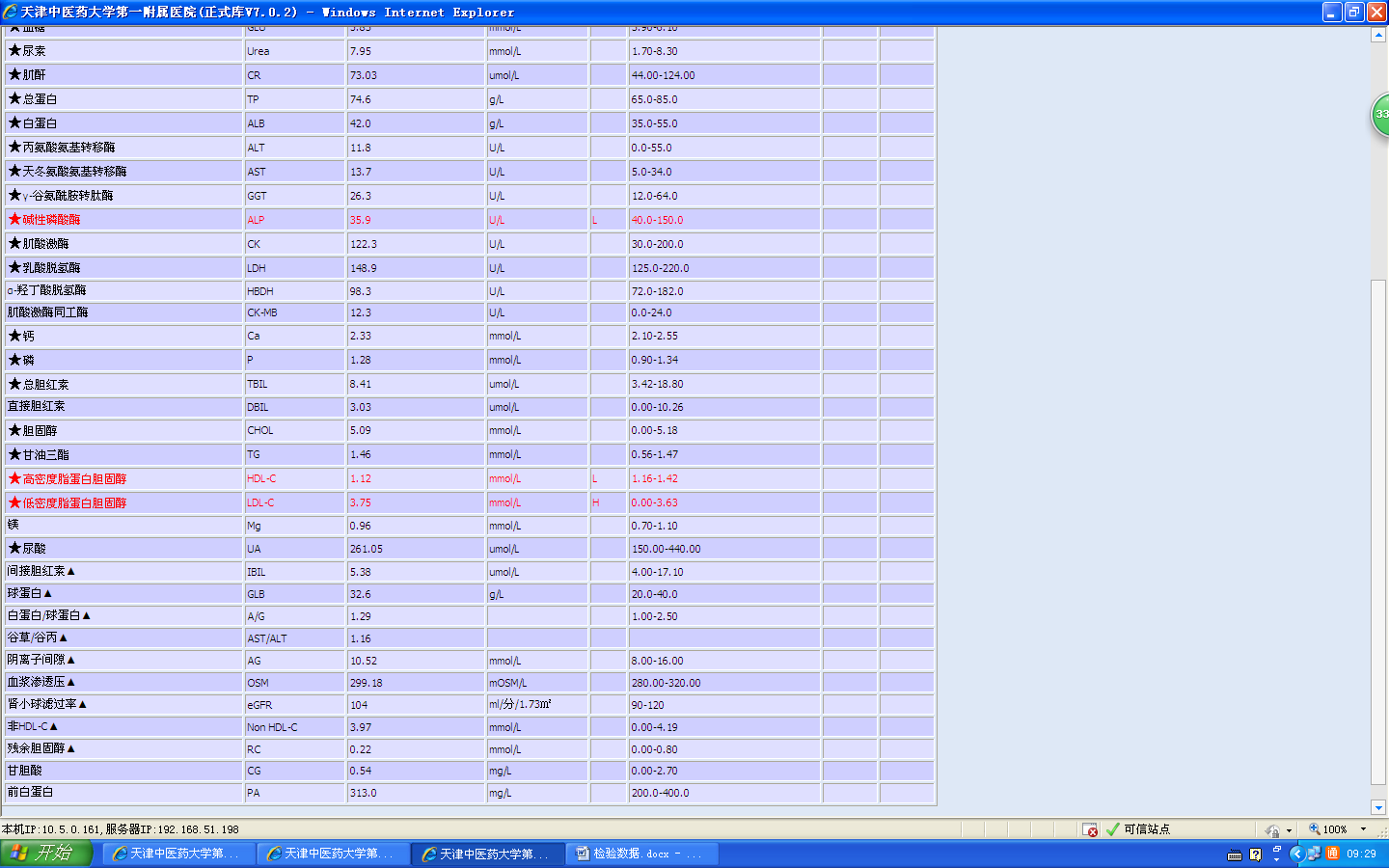


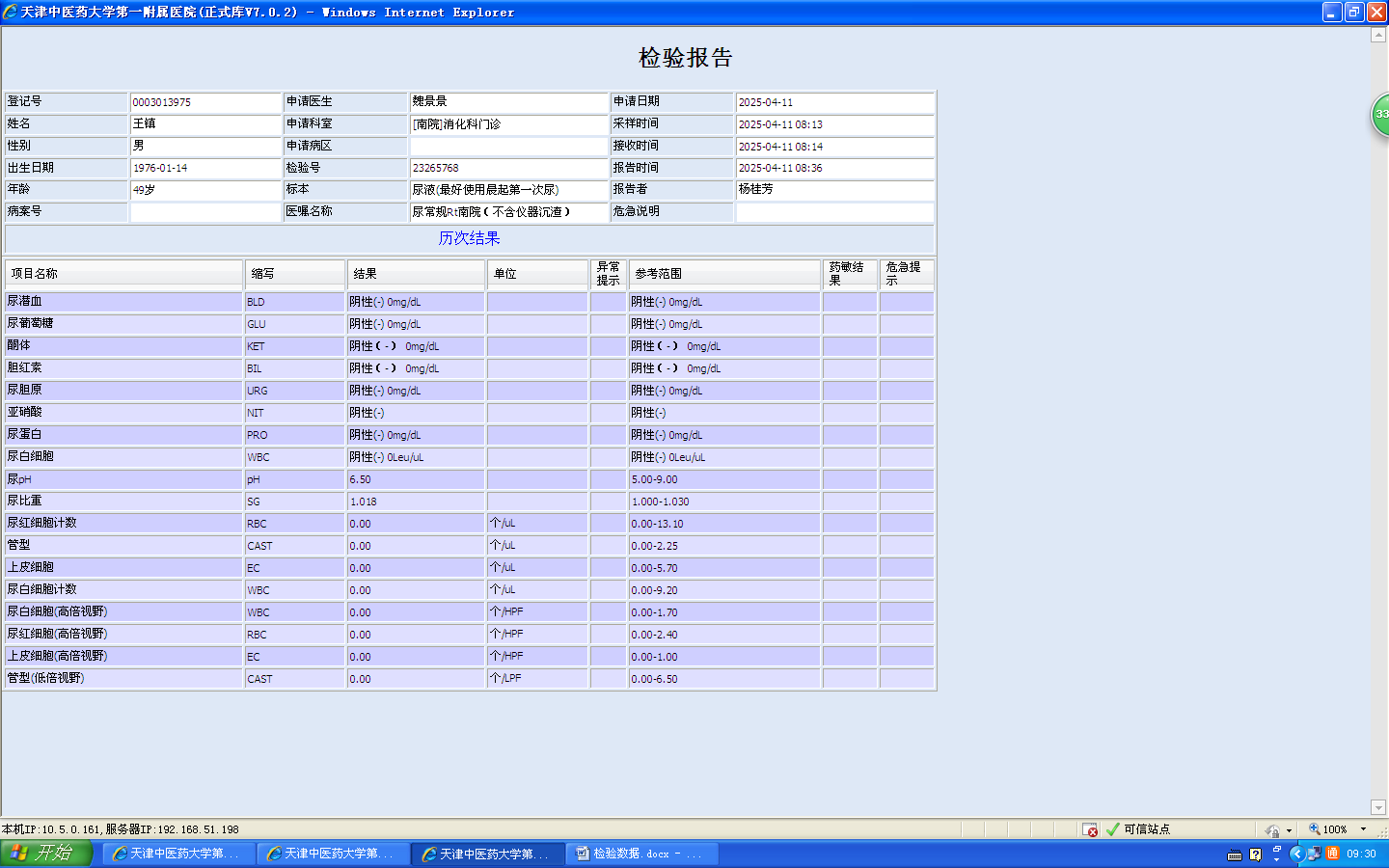

Supplement: Supplementary file 1 [file medi-105-e48157-s001.doc]
